# The l,d-transpeptidation pathway is inhibited by antibiotics of the β-lactam class in *Clostridioides difficile*

**DOI:** 10.1016/j.isci.2025.112227

**Published:** 2025-03-16

**Authors:** Ana M. Oliveira Paiva, Pascal Courtin, Glenn Charpentier, Imane Oueled-Chama, Olga Soutourina, Marie-Pierre Chapot-Chartier, Johann Peltier

**Affiliations:** 1 Université Paris-Saclay, CEA, CNRS, Institute for Integrative Biology of the Cell (I2BC), Gif-sur-Yvette, France; 2 Université Paris-Saclay, INRAE, AgroParisTech, Micalis Institute, Jouy-en-Josas, France

**Keywords:** Biochemistry, Microbiology, Pharmacology

## Abstract

The resistance of *Clostridioides difficile* to the β-lactam antibiotics cephalosporins, which target the peptidoglycan (PG) assembly, is a leading contributor to the development of *C. difficile* infections. *C. difficile* has an original PG structure with a predominance of 3→3 cross-links generated by l,d-transpeptidases (LDTs). *C. difficile* forms spores and we show that the spore cortex PG contains exclusively 3→3 cross-links. PG and spore cortex of *C. difficile* cells were largely unaffected by the deletion of the three predicted LDTs, revealing the implication of a new family of LDTs. The d,d-carboxypeptidases producing the essential LDT substrate were inactivated by cephalosporins, resulting in the inhibition of the l,d-transpeptidation pathway. In contrast, the participation of penicillin-binding proteins (PBPs) to PG cross-linking increased in the presence of the antibiotics. Our findings highlight that cephalosporin resistance is not primarily mediated by LDTs and illustrate the plasticity of the PG biosynthesis machinery in *C. difficile*.

## Highlights

• *C. difficile* relies on non-classical l,d-transpeptidases for peptidoglycan cross-linking
• The spore cortex of *C. difficile* is exclusively cross-linked by l,d-transpeptidation
• d,d-carboxypeptidases are the primary target of β-lactams in *C. difficile*
• d,d-carboxypeptidases controls the mode of transpeptidation in *C. difficile*

## Graphical abstract

**Figure:**
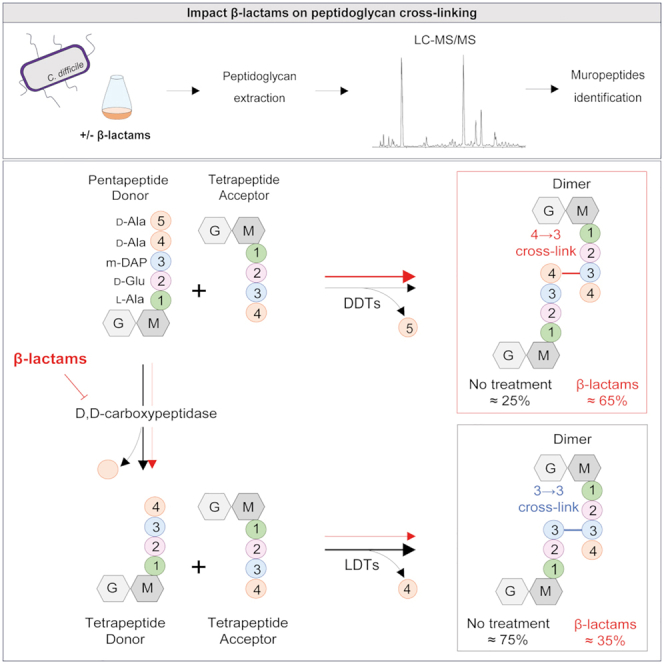

## Introduction

**Figure 1:**
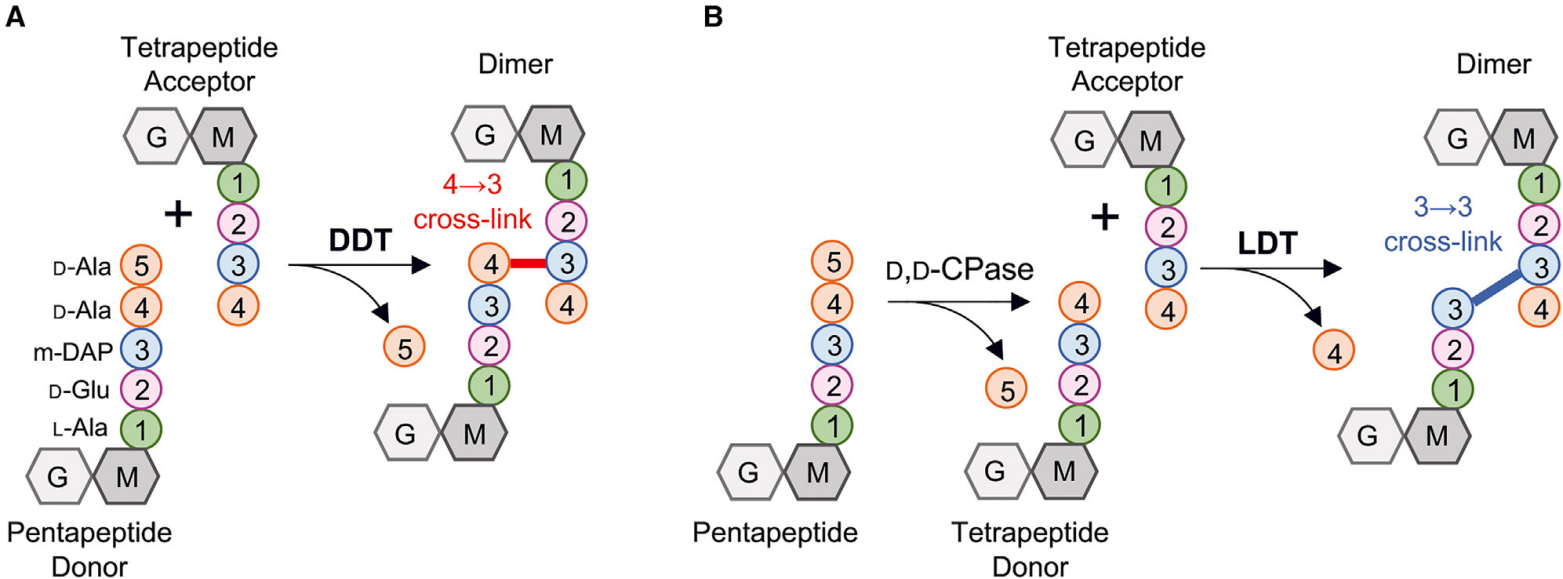
Formation of PG cross-links by the d,d- and the l,d-transpeptidation pathway (A) The d,d-transpeptidase (DDT) activity of the PBPs cleaves the terminal d-Ala of a native pentapeptide stem donor and subsequently generates a 4→3 cross-link with an acceptor stem. (B) l,d-transpeptidases (LDT) cleave the terminal d-Ala of a tetrapeptide stem donor and subsequently generate a 3→3 cross-link with an acceptor stem. Enzymes with d,d-carboxypeptidase (d,d-CPase) activity produce the essential tetrapeptide donors from pentapeptide native stems. G, *N*-acetylglucosamine; M, *N*-acetylmuramic acid.

*Clostridioides difficile* is the primary cause of antibiotic-associated nosocomial diarrhea in adults.^1^ *C. difficile* forms spores that are essential for the dissemination of *C. difficile* infections (CDI).^2^ Antibiotic exposure resulting in gastrointestinal dysbiosis is commonly associated with the development of CDI.^1^ β-lactam antibiotics, particularly cephalosporins, are widely recognized as key factors in the development of CDI. However, the mechanism underlying the intrinsic resistance of *C. difficile* to these antibiotics remains poorly understood.^3^^,^^4^ β-lactams target the cell wall peptidoglycan (PG) assembly. PG is a polymeric macromolecule consisting of linear glycan chains cross-linked by short peptides.^5^ The d,d-transpeptidase (DDT) activity of penicillin-binding proteins (PBPs) connects the fourth amino acid of one peptide side chain to the third amino acid of another, forming 4→3 cross-links (Figure 1A). However, 3→3 cross-links generated by l,d-transpeptidases (LDTs) presenting a YkuD-like domain have also been reported (Figure 1B).^6^ In addition to their cross-linking activity, both DDTs and LDTs catalyze exchange reactions where the terminal d-Ala in pentapeptides and tetrapeptides, respectively, are replaced by a non-canonical d-amino acid (NCDAA).^7^^,^^8^ DDTs, which are the primary targets of β-lactam antibiotics, use native PG precursors containing a pentapeptide stem as acyl donors for cross-linking. In contrast, LDT activity requires a tetrapeptide stem as the acyl donor substrate.^6^ This substrate is typically produced by d,d-carboxypeptidases that cleave the fifth residue from the pentapeptide chain (Figure 1B).^9^^,^^10^^,^^11^ LDTs have the potential to cf. resistance to β-lactam antibiotics, as they are solely inhibited by β-lactam antibiotics of the carbapenem class.^12^

*C. difficile* is the only known Bacillota with predominant 3→3 PG cross-links.^6^^,^^13^^,^^14^ Recent studies revealed that a mutant strain of *C. difficile* R20291, which lacks all three LDT paralogues, is not impaired in the production of 3→3 cross-links.^15^^,^^16^ Furthermore, two new enzymes with a VanW catalytic domain have been identified as the missing LDTs.^16^ However, the role of 3→3 cross-linking in mediating cephalosporin resistance in *C. difficile* remains unclear. While the structure of the *C. difficile* spore cortex, a modified layer of PG, has also been characterized, the type of cross-links present has not been defined.^17^

In this study, we showed that the spore cortex PG of *C. difficile* only contains 3→3 cross-links. As for the PG of vegetative cells, 3→3 cross-links were still present in the spore cortex of a *C. difficile* strain lacking the three YkuD domain-containing LDTs, suggesting the implication of the VanW domain proteins. By treating *C. difficile* cells with subinhibitory concentrations of β-lactams, including cephalosporins and carbapenems, we discovered that at least one d,d-carboxypeptidase, which is necessary for the production of LDT substrates, is the primary target of these antibiotics and is differentially sensitive to them*.* In contrast, PBPs generating the 4→3 cross-links are not inhibited by these concentrations of antibiotics. Thus, cephalosporin resistance in *C. difficile* is not dependent on PG cross-linking by LDTs and instead relies on the poor inhibition of PBPs by this antibiotic family. These findings suggest that the growth arrest induced by β-lactams is mediated by the inhibition of thel,d-transpeptidation pathway in *C. difficile*.

## Results

### A strain lacking the three YkuD proteins still predominantly produces 3→3 cross-links

To evaluate the role of the three proteins harboring the catalytic domain YkuD on the PG structure of *C. difficile* 630Δ*erm*, we generated a triple mutant of the corresponding genes *ldt*_*Cd1*_ (*CD2963*), *ldt*_*Cd2*_ (*CD2713*), and *ldt*_*Cd3*_ (*CD3007*) (Figures S1A and S1B).^14^ This mutant, hereby referred as ΔΔΔ*ldt*, was confirmed via whole-genome sequencing and no additional mutation could be detected (see STAR Methods). The growth rate of the ΔΔΔ*ldt* strain was similar to that of the parental strain 630Δ*erm* (Figure S1C).

**Figure 2:**
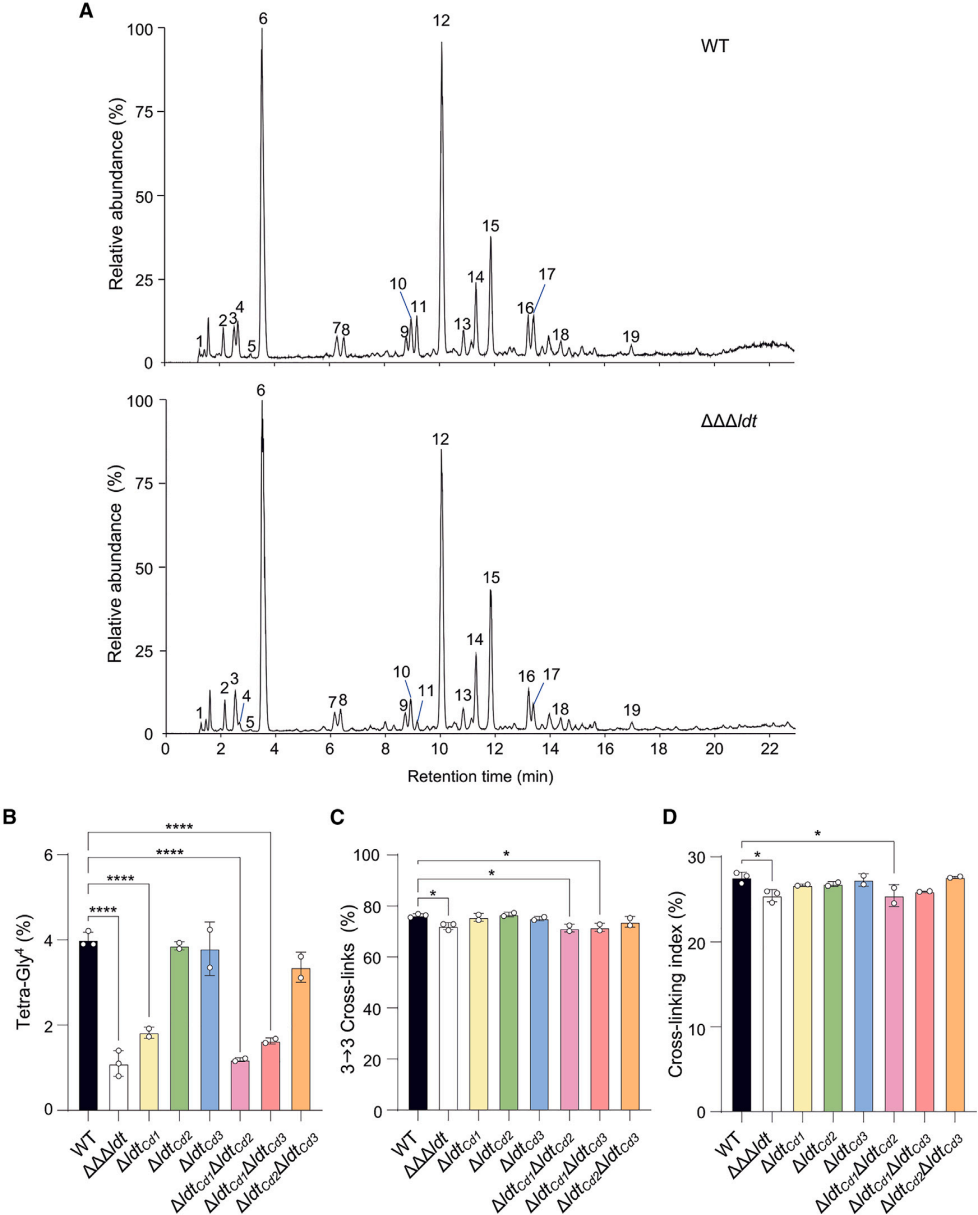
Impact of the *ldt* single, double and triple deletions on the PG structure of *C. difficile* vegetative cells (A) LC-MS chromatograms (total ion current [TIC]) of muropeptides from vegetative cells of *C. difficile* 630Δ*erm* wild-type (WT) and ΔΔΔ*ldt* strains. Major peaks are labeled with numbers referring to Table S1. See also Table S1 for the structure of all identified muropeptides. Data are representative of three independent experiments. (B) Abundance of muropeptides containing a modified tetrapeptide stem with the LDT-mediated exchange of the terminal d-Ala by a Gly in the PG from *C. difficile* WT and *ldt* mutant strains. (C) Abundance of muropeptide dimers with a 3→3 cross-link relative to the total cross-links in dimers in the PG from *C. difficile* WT and *ldt* mutant strains. (D) Cross-linking index of PG from *C. difficile* WT and *ldt* mutant strains. All graphs represent mean ± SEM and include individual data points; *n* = 3 independent experiments for WT and ΔΔΔ*ldt* and *n* = 2 independent experiments for the other mutant strains. ∗*p* ≤ 0.05 and ∗∗∗∗*p* ≤ 0.0001 by a one-way ANOVA followed by a Dunnett’s multiple comparisons test.

Wild-type and ΔΔΔ*ldt* cells were collected in exponential growth and the purified PG was digested with mutanolysin to generate muropeptides. Muropeptides were analyzed by ultra-high performance liquid chromatography coupled to tandem mass spectrometry (UHPLC-MS/MS) (Figure 2A) and their structure was deduced from their *m/z* values and fragmentation patterns obtained by MS/MS. The structure of all identified muropeptides is summarized in Table S1. The muropeptide composition of the ΔΔΔ*ldt* strain was largely similar to that of the parental strain (Figure 2A; Table S1). The main difference was a strong reduction of the abundance of muropeptides containing a tetrapeptide stem ending in Gly (peaks 4 and 11) in the mutant compared to the wild-type strain (Figures 2A and 2B; Table S1), revealing a role of at least one of the three enzymes in the exchange reaction of the terminal d-Ala in tetrapeptide stems with Gly. Additionally, a small but significant decrease of the dimers cross-linked by l,d-transpeptidation, which results in a reduction of the cross-linking index was observed in the ΔΔΔ*ldt* strain compared to the parental strain (Figures 2C and 2D). Nonetheless, 3→3 cross-links remained predominant in the triple mutant strain, indicating the presence of LDTs with a different catalytic domain compensating for the loss of the canonical LDTs.

To confirm the impact of the mutations on the exchange reaction, *C. difficile* cells were labeled with the amino acid probe HCC-amino d-alanine (HADA) during exponential growth,^18^^,^^19^ and visualized using fluorescence microscopy (Figures S2A and S2B). The cell length and width between the triple mutant and the parental strain were identical (Figures S2C and S2D). In pre-divisional cells, the intensity of the HADA staining significantly increased at mid-cell, as expected for the septum formation (Figures S2B, S2E, and S2F). In dividing cells, a gradient of fluorescence was observed with a concentrated signal at one of the poles, which presumably corresponds to the newly formed pole, and a fading signal at the other pole (Figures S2B and S2F). The triple mutant showed a similar fluorescent labeling profile to the wild type around the cells, but an overall significant decrease in fluorescence intensity (Figures S2B and S2E). This result suggests that HADA incorporation through the exchange reaction into the PG of *C. difficile* is partially dependent on the activity of at least one of the LDTs.

### Ldt_Cd1_ contributes to the exchange reaction and the generation of 3→3 cross-links

To elucidate the contribution of the three LDTs to exchange reactions and 3→3 cross-linking, the muropeptide profile of the different single and double mutants was also analyzed (Figure S3; Table S1). The abundance of tetrapeptides ending in Gly and of 3→3 cross-links in the strains deleted of *ldt*_*Cd2*_ and/or *ldt*_*Cd3*_ were similar to those of wild type (Figures 2B and 2C). However, deletion of either *ldt*_*Cd1*_, *ldt*_*Cd1*_, and *ldt*_*Cd2*_ or *ldt*_*Cd1*_ and *ldt*_*Cd3*_ recapitulated the decrease of tetrapeptides ending in Gly observed in the ΔΔΔ*ldt* strain. The proportion of 3→3 cross-links was also reduced in the Δ*ldt*_*Cd1*_Δ*ldt*_*Cd2*_ and Δ*ldt*_*Cd1*_Δ*ldt*_*Cd3*_ double mutants with an impact on the cross-linking index (Figures 2C and 2D). Thus, these data indicate that the PG modifications observed in the triple mutant strain are mostly mediated by the *ldt*_*cd1*_ deletion.

### The spore cortex of *C. difficile* is cross-linked by l,d-transpeptidation

**Figure 3:**
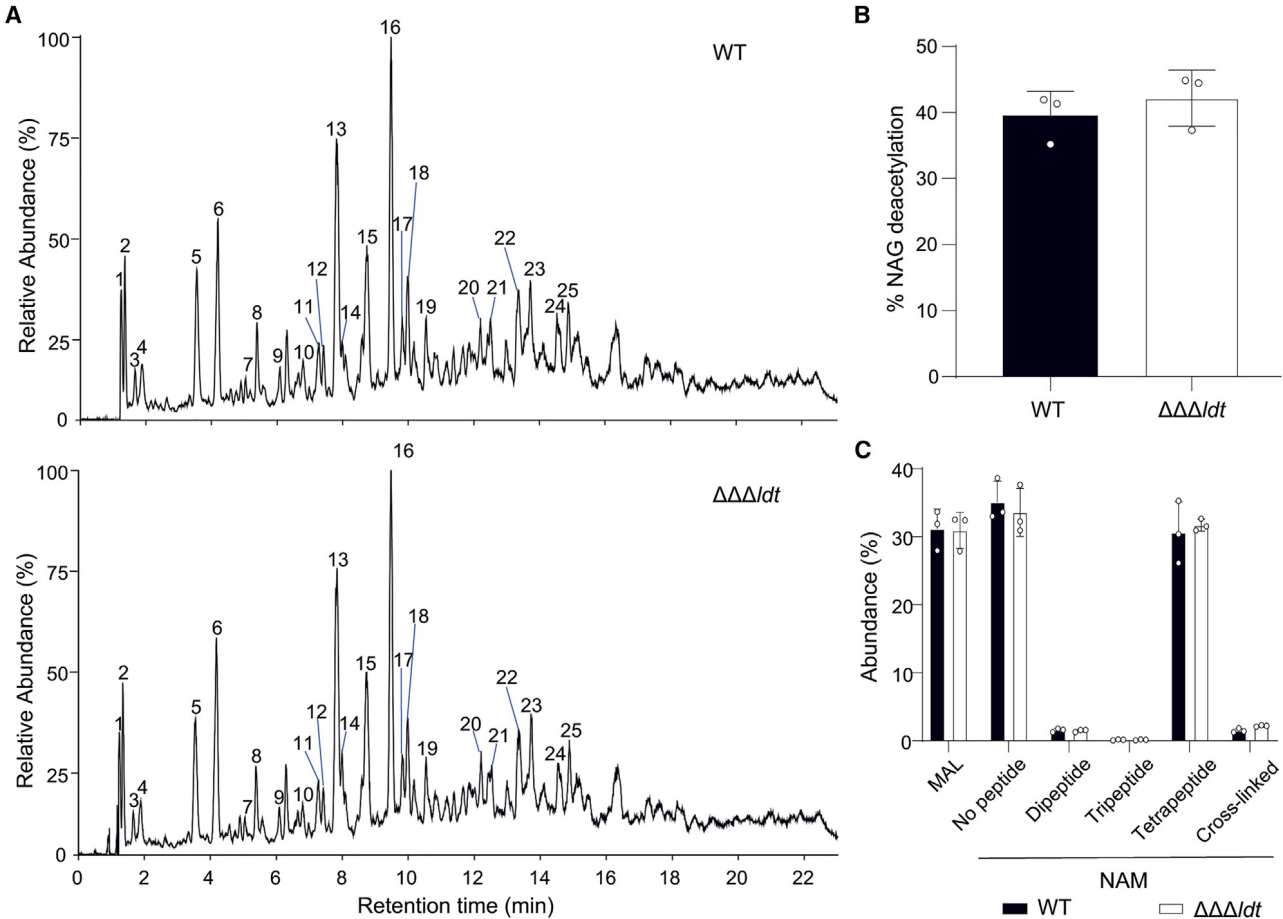
Impact of the *ldt* triple deletion on the PG structure of *C. difficile* spore cortex (A) LC-MS chromatograms (TIC) of muropeptides from the spore cortex of *C. difficile* 630Δ*erm* wild-type (WT) and ΔΔΔ*ldt* strains. Major peaks are labeled with numbers referring to Table S2. See also Table S2 for the structure of all identified muropeptides. Data are representative of three independent experiments. (B) Abundance of NAG deacetylation in the spore cortex from *C. difficile* WT and ΔΔΔ*ldt* strains. (C) Abundance of MAL and NAM in the spore cortex from *C. difficile* WT and ΔΔΔ*ldt* strains. Percentages of unsubstituted NAM (no peptide) and of NAM substituted with a dipeptide, a tripeptide or a tetrapeptide, or involved in cross-linking are represented. All graphs represent mean ± SEM and include individual data points; *n* = 3 independent experiments.

To determine the mode of cross-linking in the spore cortex, spores of *C. difficile* 630 Δ*erm* wild type were purified and their PG cortex was isolated. Muropeptides were generated using mutanolysin, analyzed by UHPLC-MS/MS (Figure 3A), and their structure was deduced from their *m/z* values and fragmentation patterns obtained by MS/MS. All the identified peaks are summarized in Table S2. In agreement with a previous report,^17^ 39.5% ± 3.7% of the NAG residues were found to be *N*-deacetylated in the spore cortex of *C. difficile* wild type (Figure 3B). In addition, 31.1 ± 2.9% of the NAM residues were converted to the spore-specific MAL in *C. difficile* spore cortex (Figure 3C). This modification prevents the cleavage between the NAM and NAG residues by mutanolysin, leading to the presence of oligosaccharides among the generated muropeptides. Most of the muropeptides still containing an unmodified NAM residue lacked any stem peptide (34.7 ± 3.1%) or harbored a tetrapeptide stem (30.6 ± 4.6%) (Figure 3C).

**Figure 4:**
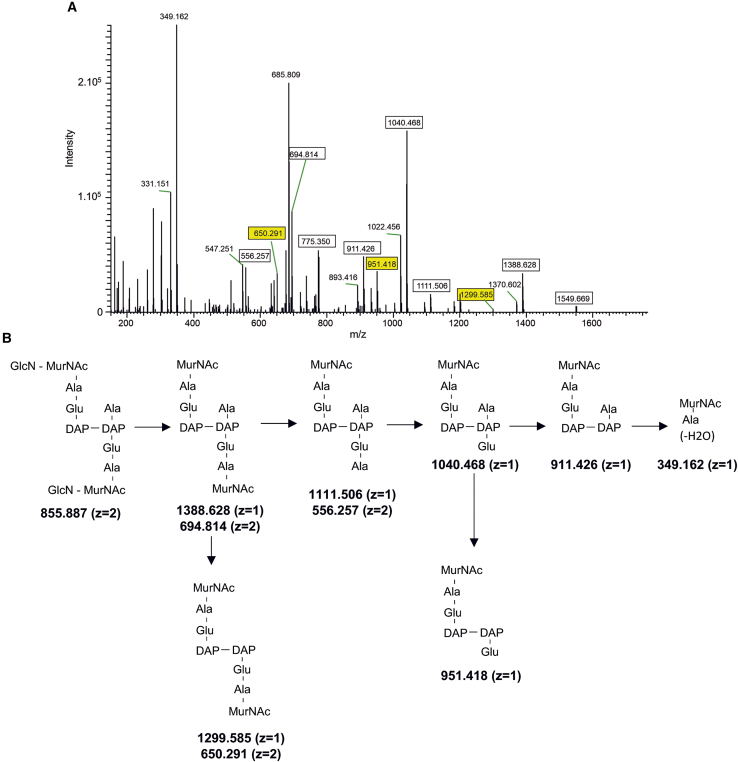
MS/MS analysis of the main muropeptide dimer from the spore cortex of *C. difficile* wild type (A) MS/MS analysis of the molecular ion [M+2H]^2+^ (*m/z* = 855.887 with *z* = 2) detected in peak 14 with a retention time of 7.99 min in Figure 4A and Table S2. Fragments (squared) were detected as [M + H]^+^ (z = 1) or [M+2H]^2+^ (z = 2) adducts. Fragments resulting from loss of a water molecule are not squared. The fragments allowing assignment of the 3-3 cross-links are highlighted in yellow. (B) Structures inferred from the MS/MS analysis are presented. The loss of one alanine from the C-terminal end of different ions (mass loss of 89.048) establishes the presence of a 3→3 cross-link.

Among the identified muropeptides, only two corresponded to dimers and no trimer was detected (Table S2). Thus, the spore cortex of *C. difficile* is very weakly cross-linked with a cross-linking index of 1.5 ± 0.3% (Figure 3C). The two dimers had the same structure with a tripeptide and a tetrapeptide stem and both contained a 3→3 cross-link as revealed by the loss of one C-terminal alanine (mass loss of 89.048 Da) from the acceptor tetrapeptide stem in their MS-MS fragmentation spectrum (Figures 4 and S4). Thus, the spore cortex of *C. difficile* contains only 3→3 cross-links.

Next, we analyzed the spore PG structure of the ΔΔΔ*ldt* strain to assess the implication of the LDTs in the formation of the 3→3 cross-links. However, the muropeptide profile of the mutant was identical to that of the wild-type strain and no decrease of the 3→3 cross-links was observed (Figure 3; Table S2). These data indicate that either the canonical LDTs do not contribute to the cross-linking of the spore cortex or their loss is compensated by the activity of at least one of the two novel LDTs with a VanW domain.^16^

### The YkuD domain proteins do not contribute to β-lactam resistance in *C. difficile*

We investigated the effect of the triple *ldt* deletion on β-lactam resistance in *C. difficile*. Loss of the three l,d-transpeptidases did not alter the minimum inhibitory concentration (MIC) of different members of the β-lactam antibiotics class (Table S3). In addition, growth of the ΔΔΔ*ldt* and the parental strains in the presence of subinhibitory concentrations of different β-lactams (1/2 MIC) were similar (Figure S5). We reasoned that the gene expression of the VanW domain LDTs or other enzymes of the PG polymerization machinery might be induced in the presence of β-lactams to compensate for the loss of the three YkuD proteins. To test this hypothesis, 630Δ*erm* and ΔΔΔ*ldt* were grown in the presence of subinhibitory concentrations of the cephalosporin antibiotic ceftriaxone (1/2 MIC) and the samples were subjected to RNA sequencing. Whereas genes involved in stress response, such as type I toxin-antitoxin systems were induced in ΔΔΔ*ldt*, no gene related to PG metabolism, including the VanW proteins (*CD1436* and *CD2149* in 630Δ*erm*) or encoding PG-associated proteins could be identified as differently expressed (Table S4). These data indicate that the YkuD domain proteins are dispensable for β-lactam resistance in *C. difficile* 630Δ*erm*.

### The l,d-transpeptidation pathway is inhibited by subinhibitory concentrations of cephalosporin and carbapenem antibiotics in *C. difficile*

**Figure 5:**
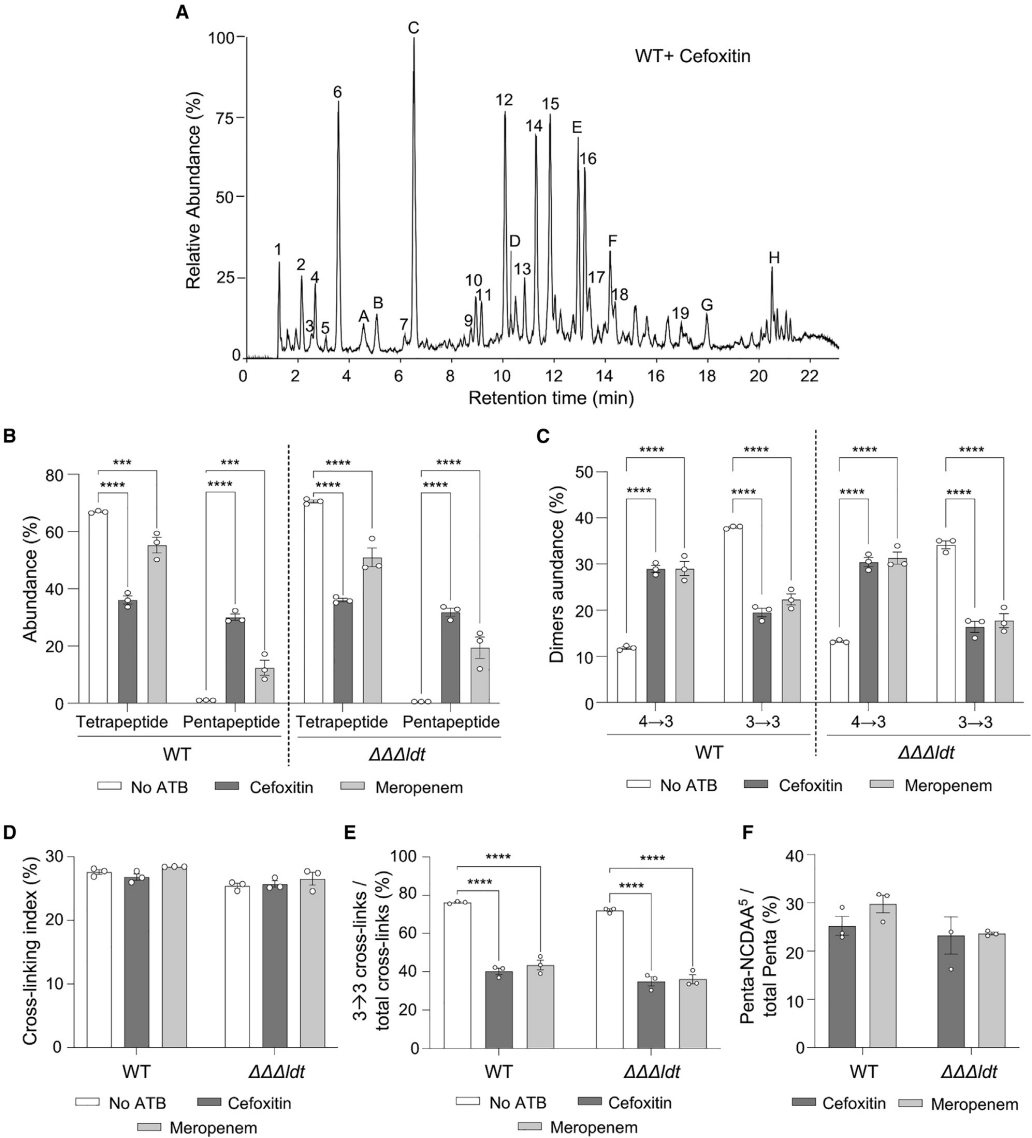
Impact of different β-lactam antibiotics on the PG structure of vegetative cells of *C. difficile* wild type and ΔΔΔ*ldt* (A) LC-MS chromatogram of muropeptides from vegetative cells of *C. difficile* wild-type (WT) strain grown in the presence of subinhibitory concentrations of cefoxitin. Peak labels refer to Table S1. New major peaks observed in the presence of the antibiotics are labeled with letters. See also Table S1 for the structure of all identified muropeptides. Data are representative of three independent experiments. (B) Abundance of muropeptides with a tetrapeptide or a pentapeptide stem (free side or acceptor chain in multimers) in the PG of *C. difficile* wild-type and ΔΔΔ*ldt* strains grown in the absence of antibiotics (no ATB) or in the presence of cefoxitin or meropenem. (C) Abundance of muropeptide dimers with a 4→3 and a 3→3 cross-link relative to total muropeptides in the PG of *C. difficile* wild-type and ΔΔΔ*ldt* strains grown in the absence of antibiotics (no ATB) or in the presence of cefoxitin or meropenem. (D) Cross-linking index of the PG of *C. difficile* wild-type and ΔΔΔ*ldt* strains grown in the absence of antibiotics (no ATB) or in the presence of cefoxitin or meropenem. (E) Abundance of muropeptide dimers with a 3→3 cross-link relative to the total cross-links in dimers in the PG of *C. difficile* wild-type and ΔΔΔ*ldt* strains grown in the absence of antibiotics (no ATB) or in the presence of cefoxitin or meropenem. (F) Abundance of muropeptides with a pentapeptide stem ending with a non-canonical d-amino-acid (NCDAA: Gly, Phe, Leu, or Val) (Penta-NCDAA^5^) relative to the total pentapeptide muropeptides in the *C. difficile* wild-type and ΔΔΔ*ldt* strains grown in the presence of cefoxitin or meropenem. All graphs represent mean ± SEM and include individual data points; *n* = 3 independent experiments. ∗∗∗*p* ≤ 0.01 and ∗∗∗∗*p* ≤ 0.001 by a two-way ANOVA followed by a Dunnett’s multiple comparisons test.

To examine the impact of β-lactams on PG cross-linking, the PG structure of vegetative cells of the wild-type strain grown in the presence of 1/2 MIC of the cephalosporin cefoxitin was analyzed. *C. difficile* exposure to this antibiotic led to drastic PG structure modifications, in comparison to the non-treated strain, with the appearance of new abundant muropeptides with a pentapeptide stem to the detriment of tetrapeptide stems (Figures 2A, 5A, and 5B; Table S1). Muropeptides with a pentapeptide stem accounted for 30 ± 1.9% of the total muropeptides in the cefoxitin-treated strain when they represented only 1 ± 0.1% in the untreated strain. Conversely, the abundance of tetrapeptides decreased from 66.9 ± 0.5% to 36.1 ± 2.5% in the presence of the antibiotic (Figures 5A and 5B; Table S1). These changes were accompanied by a strong decrease of the abundance of muropeptide dimers containing 3→3 cross-links (Figure 5C; Table S1). Remarkably, the cross-linking index remained similar in the absence and in the presence of antibiotics as the 3→3 cross-link reduction was compensated by an equivalent increase of the 4→3 cross-linked dimers (Figures 5C and 5D). Overall, the proportion of 3→3 cross-links relative to the total cross-links shifted from 76.3 ± 0.7% in the non-treated wild-type strain to 40.3 ± 2.9% in the strain treated with cefoxitin (Figure 5E). Similar results were obtained when analyzing the PG structure of the ΔΔΔ*ldt* strain grown in the presence of cefoxitin, revealing the involvement of the non-canonical LDTs in the synthesis of the residual 3→3 cross-links (Figures 5B–5E and S6A; Table S1). Moreover, treatment of the ΔΔΔ*ldt* strain with ceftriaxone, another antibiotic of the cephalosporin class, resulted in similar PG modifications, although at a lesser extent (Figures S7A–S7E). Thus, these data strongly suggest that cephalosporins inactivate at high concentrations one or several d,d-carboxypeptidase(s), whose activity is required to produce the substrate of the LDT. They also reveal that at least one PBP catalyzing the formation of the 4→3 cross-linking reaction is not inactivated by these antibiotics.

*C. difficile* 630Δ*erm* is resistant to most cephalosporins but susceptible to carbapenems (Table S3).^20^ To assess whether carbapenems also impacted the activity of d,d-carboxypeptidases in *C. difficile*, PG structure of wild-type and ΔΔΔ*ldt* cells treated with 1/2 MIC of meropenem was analyzed (Figures 5B, S6A, and S6B; Table S1). As with cephalosporins, meropenem induced the appearance of several major muropeptide peaks with pentapeptide stems and modified the balance between 4→3 and 3→3 cross-links to the benefits of 4→3 cross-links in the two strains (Figures 5B–5E). Treatment of the ΔΔΔ*ldt* strain with imipenem, another antibiotic of the carbapenem class led to the same PG modifications (Figures S7A–S7E).

Another remarkable effect of the antibiotic treatments on the PG structure was the presence of a high proportion of muropeptides with a pentapeptide stem ending with a non-canonical amino acid (NCDAA: Gly, Phe, Leu, or Val) (Table S1; Figures 5F and S7F). The incorporation of NCDAA into the PG was shown to regulate the cell wall structure in response to environmental stresses in *Vibrio cholerae* and the same mechanism was suggested in *Bacillus subtilis*.^8^^,^^21^

Altogether, these data strongly suggest that at least one d,d-carboxypeptidase plays a pivotal role in the control of the mode of transpeptidation in *C. difficile*. This d,d-carboxypeptidase is the primary target of antibiotics of the β-lactam family and is differentially susceptible to these antibiotics with an efficient inhibition by low concentrations of carbapenems but only by high concentrations of cephalosporins. The associated reduction of 3→3 cross-linking, resulting from the limited LDT substrate availability, highlights that cephalosporin resistance is not primarily mediated by LDTs in *C. difficile* but rather relies on PBPs resistant to this class of antibiotics.

## Discussion

In agreement with recent studies,^15^^,^^16^ we showed here that a *C. difficile* 630Δ*erm* strain lacking the three predicted LDTs presented only a slight reduction of 3→3 cross-links and a decrease in the exchange reaction. The two novel LDTs with a VanW domain most likely compensate for the loss of the other enzymes as recently shown in the strain R20291.^16^ PG analysis of the different *ldt* single and double mutant strains further identified Ldt_Cd1_ as the enzyme mediating most of the observed changes. This result is contrasting with *in vitro* assays of the three recombinant LDTs showing that Ldt_Cd1_ is poorly active and has a weak activity in catalysis of the exchange reaction in comparison to Ldt_Cd2_ and Ldt_Cd3_.^15^^,^^22^

We showed the abundant presence of muramic-δ-lactams and *N*-acetylmuramic acids with no peptide stem, which results in a low amount of cross-links, in the spore cortex of *C. difficile*. Furthermore, we discovered that 3→3 cross-links are largely predominant, if not exclusive, in the spore cortex. In *B*. *subtilis*, the enzymes required for the cross-linking of the spore cortex are produced in the mother cell and their expression is under control of SigE,^23^ a sporulation-specific sigma factor. Previous transcriptomic analyses identified *ldt*_*Cd3*_ gene as a member of the SigE regulon in *C. difficile*.^24^^,^^25^^,^^26^ In addition, Ldt_Cd3_ was shown to display a strong LDT activity *in vitro*.^15^^,^^22^ Yet, deletion of the three LDT-encoding genes had no impact on the abundance of the cross-links in the cortex PG, implying the involvement of the non-canonical LDTs. Future work will be required to identify which LDTs contribute to the formation of the spore cortex PG.

Among the inhibitors of PG polymerization, β-lactam antibiotics stand out as the most clinically significant. The β-lactam ring found in all β-lactam antibiotics interacts with the essential nucleophilic serine of PBPs, which can display DDT, endopeptidase, or d,d-carboxypeptidase activity.^27^ This interaction results in the creation of a stable acyl-enzyme adduct that interferes with catalysis. In contrast, LDTs are slowly acylated by β-lactams of the penicillin and cephalosporin classes and the resulting acyl-enzymes are rapidly hydrolyzed.^28^ Thus, LDTs are insensitive to these antibiotics but are still efficiently inactivated by carbapenems.^29^^,^^30^ In agreement, activation of the l,d-transpeptidation pathway in *Enterococcus faecium* and *Escherichia coli* conferred broad-spectrum resistance to β-lactams, with the exception of carbapenems.^9^^,^^29^ Conversely, we showed that this pathway is inhibited by both cephalosporins and carbapenems in *C. difficile*, revealing that it does not mediate resistance to these drugs. An inhibition of the DDT activity of PBPs could explain the accumulation of muropeptides with a pentapeptide stem observed in the presence of the antibiotics. However, this hypothesis is not supported by our data as 4→3 PG cross-links were more abundant in antibiotic-treated cells. In line with this result, biochemical analyses of the purified class A PBP1 and the class B PBP2 of *C. difficile*, which are respectively essential for cross-links synthesis during vegetative cell division and elongation, revealed that both enzymes are insensitive to most cephalosporins and carbapenems.^31^^,^^32^ The only other explanation to the observed structural changes is the inhibition of at least one d,d-carboxypeptidase. Activation of the l,d-transpeptidation pathway leading to β-lactam resistance in *E. faecium* and *E. coli* requires the production of a β-lactam-insensitive d,d-carboxypeptidase to generate the tetrapeptide substrate of LDTs.^9^^,^^11^ In contrast, production of the essential LDT-tetrapeptide substrate by a β-lactam-sensitive d,d-carboxypeptidase in *C. difficile* results in the suppression of LDT activity in the presence of the antibiotics. Altogether, our data support the hypothesis that at least one d,d-carboxypeptidase, controlling substrate availability for LDTs, is the primary target for cephalosporins and carbapenems in *C. difficile*. Since 3→3 cross-links are essential for viability in this bacterium,^16^ substrate deprivation for LDTs most likely represents the original mechanism by which cephalosporins and carbapenems impact *C. difficile* survival. *C. difficile* 630 encodes 4 putative β-lactam-sensitive d,d-carboxypeptidase belonging to the PBP family (pfam00768) with two of them being required for formation of heat-resistant spores.^33^ Future research will seek to identify which d,d-carboxypeptidase is involved, as this enzyme represents an attractive target for the development of new therapies to fight *C. difficile* infections.

### Limitations of the study

Despite its significant implications, our study has several limitations. First, this investigation was conducted using only the laboratory strain 630Δ*erm* of *C. difficile* as a model. While the primary findings of this study are likely applicable to clinical strains of *C. difficile*, this remains to be experimentally demonstrated. Second, our study strongly suggests that an enzyme with a d,d-carboxypeptidase activity and belonging to the PBP family is the killing target of β-lactams in *C. difficile*. However, further research is required to explore the impact of a broader range of β-lactam antibiotics on the PG structure and identify the gene encoding this d,d-carboxypeptidase. Lastly, subsequent studies are needed to investigate the reasons for the essentiality of the l,d-transpeptidation pathway in *C. difficile*.

## Resource availability

### Lead contact

Further information and requests for resources and reagents should be directed to and will be fulfilled by the lead contact, Johann Peltier (johann.peltier@i2bc.paris-saclay.fr).

### Materials availability

Strains generated in this study are available from the lead contact without restrictions.

### Data and code availability

• LC-MS/MS datasets have been deposited in the GLYCOPOST repository: GPST000426. Accession number is listed in the key resources table. RNA-seq and whole genome sequencing data have been deposited to the NCBI Sequence Read Archive (SRA). Accession number is listed in the key resources table.
• This paper does not report original code.
• Any additional information required to reanalyze the data reported in this paper is available from the lead contact upon request.

## Acknowledgments

We are grateful to Alain Guillot (Micalis, INRAE) and François Fenaille (CEA, Université Paris-Saclay) for helpful discussions. We acknowledge ChemSyBio team (Micalis, INRAE, Jouy-en-Josas) for access to LC-MS/MS facilities. The present work has benefited from Imagerie-Gif core facility supported by the Agence Nationale de la Reherche (ANR-11-EQPX-0029/Morphoscope, ANR-10-INBS-04/FranceBioImaging, and ANR-11-IDEX-0003-02/ Saclay Plant Sciences). This work was funded by the Agence Nationale de la Recherche (ANR-20-CE15-0003-DIFFICROSS to J.P.).

## Author contributions

Conceptualization, A.M.O.P., M.-P.C.-C., and J.P.; methodology, A.M.O.P. and J.P.; investigation, A.M.O.P., G.C., P.C., and I.O.-C.; writing – original draft, A.M.O.P. and J.P.; writing – review & editing, A.M.O.P., O.S., M.-P.C.-C., and J.P.; visualization, A.M.O.P., P.C., M.-P.C.-C., and J.P.; supervision, A.M.O.P. and J.P.; funding acquisition, J.P.

## Declaration of interests

The authors declare no competing interests.

## STAR★Methods

### Key resources table

**Table:** 

REAGENT or RESOURCE	SOURCE	IDENTIFIER
**Bacterial strains**		

*E. coli* NEB10β	New England Biolabs	C3019H
*E. coli* HB101(RP4)	Laboratory stock	N/A
*C. difficile* 630Δ*erm*	Laboratory stock, Hussain et al.^34^	N/A
*C. difficile* CD572: 630Δ*erm* Δ*CD2713* Δ*CD2963* ΔCD3007 (ΔΔΔ*ldt*)	This paper	N/A
*C. difficile* CD475 : 630Δ*erm* Δ*CD2963* (*ldt*_*CD1*_)	This paper	N/A
*C. difficile* CD544 : 630Δ*erm* Δ*CD3007* (*ldt*_*CD3*_)	This paper	N/A
*C. difficile* CD545 630Δ*erm* Δ*CD2713* (*ldt*_*CD2*_)	This paper	N/A
*C. difficile* CD565 630Δ*erm* Δ*CD2713* Δ*CD2963*	This paper	N/A
*C. difficile* CD561 630Δ*erm* Δ*CD2713* Δ*CD3007*	This paper	N/A
*C. difficile* CD547 630Δ*erm* Δ*CD2963* Δ*CD3007*	This paper	N/A

**Chemicals, peptides, and recombinant proteins**		

Brain heart infusion broth	BD	237500
Bacto peptone	BD	211677
Protéose peptone n° 2	Gibco	212120
Tris-base	Merck	T6687
Ammonium sulfate	Merck	A4418
LB broth	Merck	L3022
Chloramphenicol	Merck	C0378
Ampicillin	Merck	A9518
Thiamphenicol	Merck	T0261
NEBuilder HiFi DNA Assembly	New England Biolabs	E2621X
Oxoid *Clostridioides difficile* Selective supplement	Thermo Scientific	SR0096E
NucleoSpin Micobial DNA Mini Kit	Macherey-Nagel	740235.50
HADA	Tocris Bioscience	6647
HistoDenz	Merck	D2158
Cefoxitin sodium salt	Thermo Scientific	455280050
Ceftriaxone sodium salt	Thermo Scientific	455420050
Meropenem	Thermo Scientific	462810010
Imipenem	Thermo Scientific	455322500
Amoxicillin	Merck	A8523
Oxacillin sodium salt	Thermo Scientific	455440010
Urea	Thermo Scientific	A12360.36
Tris-HCl	Euromedex	EU0011-C
Sodium dodecyl sulfate 20%	Euromedex	EU0460-B
DTT	Euromedex	EU0006-B
Desoxyribonuclease I (DNase I)	Merck	D4527
Ribonuclease A (RNase)	Merck	R5503
Lipase	Merck	62301
Pronase	Roche	10165921001
Trypsin	Merck	T0303
Hydrofluoric acid 48%	Merck	1.00334.0500
Mutanolysin	Merck	M9901
Sodium borohydride	Sigma-Aldrich	452882
Ammonium formate	Fluka	17843
Methanol HPLC LC-MS grade	VWR Chemicals	83638
Qubit™ RNA High Sensitivity	ThermoScientifc	Q32852
PmeI	New England Biolabs	R0560
RNAPro solution	MP Biomedicals	116055050
Chloroform:Isoamyl alcohol 24:1	Merck	C0549
Absolute ethanol	VWR	20821.296
UltraPure DNase/RNase-Free Distilled Water	Invitrogen	10977023
TURBO DNA-free Kit	Invitrogen	AM1907

**Oligonucleotides**		

See Table S5		

**Recombinant DNA**		

pMSR0	Peltier et al.^35^	N/A
p226: pMSR0 derivative for *CD2963* (*ldt*_*CD1*_) deletion	This study	N/A
p227: pMSR0 derivative for *CD2713* (*ldt*_*CD2*_) deletion	This study	N/A
p261 pMSR0 derivative for *CD3007* (*ldt*_*CD3*_) deletion	This study	N/A

**Deposited data**		

LC-MS/MS data	GLYCOPOST repository	GPST000426
RNA-seq data	NCBI SRA	PRJNA1107170
Whole genome sequencing data	NCBI SRA	PRJNA1107170

**Software and algorithms**		

Galaxy Europe	Galaxy^36^	https://usegalaxy.eu/
Galaxy Pasteur	Galaxy^36^	https://galaxy.pasteur.fr/
MicrobeJ v 5.13I	Rueden et al.,^37^ Ducret et al.^38^	https://www.microbej.com/
CorelDRAW X8	CorelDraw	https://www.coreldraw.com/fr/
Thermo Xcalibur	ThermoFisher	https://www.thermofisher.com/fr/fr/home.html
Skyline open-source software	Pinot et al.^39^	https://skyline.ms/project/home/begin.view?/

### Experimental models and study participant details

#### Bacterial strains and growth conditions

*C. difficile* strain 630Δ*erm*, a commonly used spontaneous erythromycin-sensitive derivative of the strain 630, and mutant strains Δ*ldt*_*Cd1*_, Δ*ldt*_*Cd2*_, Δ*ldt*_*Cd3*_, Δ*ldt*_*Cd1*_Δ*ldt*_*Cd2*_, Δ*ldt*_*Cd1*_Δ*ldt*_*Cd3*_, Δ*ldt*_*Cd2*_Δ*ldt*_*Cd3*_ and Δ*ldt*_*Cd1*_Δ*ldt*_*Cd2*_Δ*ldt*_*Cd3*_ (ΔΔΔ*ldt*) were grown in an anaerobic Jacomex workstation with an atmosphere of 5% H_2_, 5% CO_2_ and 90% N_2_. *C. difficile* strains were cultured in Brain Heart Infusion broth (BHI) or Sporulation Medium for *Clostridium difficile* (SMC, 9% Bacto peptone, 0.5% proteose peptone, 0.15% Tris base, 0.1% ammonium sulphate).

Plasmids were maintained in *E. coli* strain NEB10β and transformed using standard procedures. *E. coli* HB101 carrying the plasmid RP4 was used for plasmid conjugation with *C. difficile* strains.^40^ The *E. coli* strains were cultured in Luria Bertani broth (LB) supplemented with chloramphenicol at 15 μg/mL or 100 μg/mL ampicillin when required, and were grown aerobically at 37°C.

The growth was followed by optical density reading at 600 nm. Growth curves were performed in 96-well plates in BHI medium in the absence or the presence of antibiotics (1/2 MIC), with a starting culture at a normalized OD_600_ ≈ 0.05, under anaerobic conditions at 37°C, in Stratus Plate Reader (Cerillo). The OD_600_ value for each well was recorded every 30 min.

### Method details

#### Construction of the C. difficile ldt mutants

All oligonucleotides used in this study are listed in Table S5. The *C. difficile ldt* deletion mutants were created using a toxin-mediated allele exchange method.^35^ Briefly, ∼800 bp homology arms flanking the region to be deleted were amplified by PCR from *C. difficile* 630Δ*erm*. Purified PCR products were cloned into the PmeI site of the pseudo-suicide allele-coupled exchange (ACE) vector pMSR0 using NEBuilder HiFi DNA Assembly (New England Biolabs). The resulting plasmids, listed in the key resources table, were transformed into *E. coli* strain NEB10β (New England Biolabs) and all inserts were verified by sequencing. Plasmids were then transformed into *E. coli* HB101(RP4) and transferred by conjugation into the appropriate *C. difficile* strains. Transconjugants were selected on BHI supplemented with *Clostridioides difficile* Selective Supplement (CDSS; Oxoid), and 7.5 μg/mL thiamphenicol. Allelic exchange was performed as described.^35^ All strains were confirmed by locus amplification. The triple mutant was further confirmed by whole-genome sequencing.

#### Whole genome sequencing

*C. difficile* strains were grown in BHI for 24h. Cells were harvested and genomic DNA was isolated using NucleoSpin Microbial DNA Mini kit for DNA (Macherey-Nagel). Bacterial genomes were sequenced at Plasmidsaurus (https://www.plasmidsaurus.com/) using the long-read sequencing technology from Oxford Nanopore Technologies (ONT). Using Galaxy Europe, quality controls were performed with FastQC and nanoplot and adapter trimming was performed with Porechop. The resulting reads were mapped to the reference genome using Map with minimap2. Small indels (<50 bp) and SNPs were identified using Clair3, bcftools norm and SnpSift Filter. Large indels were identified using CuteSV. Raw sequence files were deposited to the NCBI Sequence Read Archive (SRA) BioProject PRJNA1107170.

#### Fluorescence microscopy

The sample preparation for fluorescence microscopy was carried out under anaerobic conditions. *C. difficile* strains were cultured in BHI. When required, 1 mL culture was incubated with HADA at a final concentration of 10 μM (λ_em_ ≈ 450 nm, Tocris Bioscience)^19^ for 10 min and washed 3X with anaerobic PBS. Cells were spotted on GeneFrame slides with 1.5% agarose patches. Microscopy was carried out with spinning disk confocal microscopy, using an Inverted Eclipse Ti-E (Nikon) equipped with CSU-X1-A1, Nipkow Spinning Disk confocal system (Yokogawa) and ORCA-Flash4.0 LT CMOS camera (Hamamatsu). Data and statistical analysis were done with MicrobeJ version 5.13I plugin for ImageJ.^37^^,^^38^ Recognition of cells was limited to 1 μm^2^ minimum and 1 - 16 μm length. Cells with defective detection were excluded from analysis. Fluorescent intensity profiles of contour and medial were used for analysis of 2 independent biological replicates. Representative pictures were prepared for publication in CorelDRAW X8 (Corel).

#### Spore purification

Overnight cultures of *C. difficile* ΔΔΔ*ldt* and parental strains grown in BHI were spread out on twelve SMC agar plates for sporulation.^41^ After 7 days of anaerobic incubation at 37°C, cells and spores were harvested in 2 ml of ice-cold sterile water. Crude suspensions were washed 10 times with ice-cold sterile water and spores were purified with a HistoDenz gradient 20-50%.

#### Purification and structural analysis of PG

Vegetative PG was extracted from *C. difficile* strains as previously described with some modifications.^14^ *C. difficile* cultures were grown to OD_600_ ≈ 1.0 at 37°C in BHI with the addition of cefoxitin (64 μg/mL), ceftriaxone (32 μg/mL), meropenem (1 μg/mL) or imipenem (2 μg/mL), when required. Cells were harvested by centrifugation at 5000g for 10 min and processed in a FastPrep apparatus (MP Bioscience) for 30 s at 4 m/s to break the cells. For spore PG extraction, purified spores were additionally incubated twice in decoating buffer (50 mM Tris-HCl pH8.0, 8M Urea, 1% sodium dodecyl sulfate (SDS), 50 mM DTT) for 1h at 37°C. Samples were harvested by centrifugation at 17000g for 10 min.

Both cell and spore pellets were resuspended in cold H_2_O, boiled for 10 min, cooled again, and centrifuged at 17000g for 10 min. The cells were then boiled in 10% SDS, followed by boiling in 4% SDS and washed 10 times with H_2_O. The insoluble material was then treated with pronase for 90 min at 60°C, followed by incubation with DNase, RNase, Lipase and trypsin for 20h at 37°C to purify the cell wall. Finally, the samples were incubated in 48% hydrofluoric acid at 4°C for 16 h to remove wall polysaccharides. Purified PG was digested with mutanolysin (Sigma), and the soluble muropeptides were reduced with sodium borohydride. Muropeptides were analyzed by LC-MS/MS with an UHPLC instrument (Vanquish Flex, Thermo Scientific) connected to a Q-Exactive Focus mass spectrometer (Thermo Fisher Scientific) fitted with an H-ESI electrospray source (facilities located at ChemSyBio, Micalis, INRAE, Jouy-en-Josas). They were separated by reverse phase chromatography with a ZORBAX Eclipse Plus C18 RRHD column (100 by 2.1mm; particle size, and 1.8μm; Agilent Technologies) at 50°C using 10 mM ammonium formate buffer (pH 4.6) and a 20 min linear gradient from 0 to 20% methanol at a flow rate of 0.3 ml/min. Mass analysis was performed in positive mode with an acquisition range of 380 -1400 *m/z* at resolution 17,500. The Q-Exactive mass spectrometer was operated with capillary voltage at 3.5 kV and a capillary temperature set at 320°C. MS2 was performed in an acquisition range of 160-1600 m/z with an AGC target at 2.10^5^ with HCD collision anode at energy 25. Data were acquired with the Qual Browser suite (Thermo Xcalibur).

Muropeptides were identified from their *m/z* values and MS/MS spectra when required. Their relative amount was determined using Skyline open-source software according to the Small Molecule Quantification workflow.^39^ A transition list was created manually containing all the muropeptides identified by their retention times and including the different adducts and charge states detected by MS. Extraction ion chromatograms (EICs) and peak integration were realized after importing raw data from Xcalibur files. The relative amount (%) of each muropeptide was calculated as the ratio between the sum of EIC areas assigned to the muropeptide and the sum of EIC areas assigned to all identified muropeptides.

The cross-linking index (CI) was calculated according to Glauner as follows: CI = (1/2 Σdimers + 2/3 Σtrimers)/Σall muropeptides.^42^ The relative amount of muropeptides with a certain peptide side chain (X= Di, Tri, Tetra, Penta) with a free terminal carboxyl group (acceptor chain in multimers) was calculated according to Glauner as follows: percentage (X) = (Σmonomers(X) + 1/2Σdimers + 1/3Σtrimers)/Σall muropeptides.^42^

#### Minimum inhibitory concentration (MIC) determination

For the determination of the MIC, strains were grown until OD_600_ of 1.0 and inoculated to a starting optical density 0.05 in 200 μL BHI medium supplemented with the required antibiotic range (amoxicillin, oxacillin, cefoxitine, ceftriaxone, imipenem or meropenem) on 96-well plates. Cultures were incubated at 37°C for 24h. MICs for each strain were determined as the lowest concentration without visible growth, at least in three independent experiments.

#### RNA sequencing

For total RNA extraction, *C. difficile* strains were grown in 20 mL BHI medium with 32 μg/mL ceftriaxone, until an optical density of 1.0. Total RNA was isolated as previously described.^43^ Quantification of the RNA samples was performed using the Qubit™ RNA High Sensitivity (ThermoScientifc). Samples were sent for RNA sequencing by Novogene Prokaryotic RNA Sequencing services (Novogene). Analysis of the raw FastQ files was performed with Galaxy Pasteur.^36^ Quality control of the raw data was assessed using FastQC and raw data were then subjected to trimming and filtering with AlienTrimmer.^44^ Sequencing reads were aligned to *C. difficile* strain 630 genome (NC_009089.1) with the Bowtie2 software using default parameters.^45^ SARTools DESeq2 was used to perform normalization and differential analysis using values of the 630Δ*erm* wild-type strain as a reference for reporting the expression data of the ΔΔΔ*ldt* strain. Genes were considered differentially expressed if they had a ≥two-fold increase or decrease in expression and an adjusted *P* value (*q* value) ≤ 0.05. Assignment of the functional orthologues (K number) and KEGG pathway was performed automatically using BlastKOALA,^46^ and was then manually edited. RNA-seq raw sequence reads were deposited to the NCBI Sequence Read Archive (SRA) BioProject PRJNA1107170.

### Quantification and statistical analyses

Statistical analyses were performed using GraphPad Prism 10. Data are presented as the mean values ± SEM of at least three independent experiments with the following exceptions: in the Figures 2B–2D, graphs represent mean ± SEM of two independent experiments for the mutant strains Δ*ldt*_*Cd1*_, Δ*ldt*_*Cd2*_, Δ*ldt*_*Cd3*_, Δ*ldt*_*Cd1*_Δ*ldt*_*Cd2*_, Δ*ldt*_*Cd1*_Δ*ldt*_*Cd3*_, Δ*ldt*_*Cd2*_Δ*ldt*_*Cd3*_, and in the Figures S2C–S2F, two independent experiments were analysed for *C. difficile* 630*Δerm* and ΔΔΔ*ldt*. All the statistical details of experiments, including the number of independent replicates n, what n represents and the statistical tests used, can be found in the figure legends. Statistical analyses were performed using one-way ANOVA or two-way ANOVA, both followed by a Dunnett’s multiple comparisons test. For all figures, *P* values are represented as follows: ^∗^*P* < 0.05, ^∗∗^*P* < 0.01, ^∗∗∗^*P* < 0.001, ^∗∗∗∗^*P* < 0.0001.

## Supplemental information

Document S1. Figures S1–S7 and Tables S2–S5

Table S1. Structures, molecular masses and proportions of muropeptides from vegetative cells of *C. difficile* 630Δerm wild-type (WT) and mutant strains in the absence and in the presence of subinhibitory concentrations of antibiotics as indicated

## References

[1] Smits W.K., Lyras D., Lacy D.B., Wilcox M.H., Kuijper E.J. (2016). *Clostridium difficile* infection. Nat. Rev. Dis. Primers.

[2] Paredes-Sabja D., Shen A., Sorg J.A. (2014). *Clostridium difficile* spore biology: sporulation, germination, and spore structural proteins. Trends Microbiol..

[3] Gerding D.N. (2004). Clindamycin, cephalosporins, fluoroquinolones, and *Clostridium difficile*-associated diarrhea: this is an antimicrobial resistance problem. Clin. Infect. Dis..

[4] Spigaglia P. (2016). Recent advances in the understanding of antibiotic resistance in *Clostridium difficile* infection. Ther. Adv. Infect. Dis..

[5] Vollmer W., Blanot D., de Pedro M.A. (2008). Peptidoglycan structure and architecture. FEMS Microbiol. Rev..

[6] Aliashkevich A., Cava F. (2022). LD-transpeptidases: the great unknown among the peptidoglycan cross-linkers. FEBS J..

[7] Lupoli T.J., Tsukamoto H., Doud E.H., Wang T.S.A., Walker S., Kahne D. (2011). Transpeptidase-mediated incorporation of D-amino acids into bacterial peptidoglycan. J. Am. Chem. Soc..

[8] Cava F., de Pedro M.A., Lam H., Davis B.M., Waldor M.K. (2011). Distinct pathways for modification of the bacterial cell wall by non-canonical D-amino acids. EMBO J..

[9] Hugonnet J.E., Mengin-Lecreulx D., Monton A., den Blaauwen T., Carbonnelle E., Veckerlé C., Brun Y.V., van Nieuwenhze M., Bouchier C., Tu K. (2016). Factors essential for L,D-transpeptidase-mediated peptidoglycan cross-linking and beta-lactam resistance in *Escherichia coli*. Elife.

[10] Vermassen A., Leroy S., Talon R., Provot C., Popowska M., Desvaux M. (2019). Cell Wall Hydrolases in Bacteria: Insight on the Diversity of Cell Wall Amidases, Glycosidases and Peptidases Toward Peptidoglycan. Front. Microbiol..

[11] Sacco E., Hugonnet J.E., Josseaume N., Cremniter J., Dubost L., Marie A., Patin D., Blanot D., Rice L.B., Mainardi J.L., Arthur M. (2010). Activation of the L,D-transpeptidation peptidoglycan cross-linking pathway by a metallo-D,D-carboxypeptidase in *Enterococcus faecium*. Mol. Microbiol..

[12] Mora-Ochomogo M., Lohans C.T. (2021). beta-Lactam antibiotic targets and resistance mechanisms: from covalent inhibitors to substrates. RSC Med. Chem..

[13] Bern M., Beniston R., Mesnage S. (2017). Towards an automated analysis of bacterial peptidoglycan structure. Anal. Bioanal. Chem..

[14] Peltier J., Courtin P., El Meouche I., Lemée L., Chapot-Chartier M.P., Pons J.L. (2011). *Clostridium difficile* has an original peptidoglycan structure with a high level of *N*-acetylglucosamine deacetylation and mainly 3-3 cross-links. J. Biol. Chem..

[15] Galley N.F., Greetham D., Alamán-Zárate M.G., Williamson M.P., Evans C.A., Spittal W.D., Buddle J.E., Freeman J., Davis G.L., Dickman M.J. (2024). *Clostridioides difficile* canonical L,D-transpeptidases catalyze a novel type of peptidoglycan cross-links and are not required for beta-lactam resistance. J. Biol. Chem..

[16] Bollinger K.W., Müh U., Ocius K.L., Apostolos A.J., Pires M.M., Helm R.F., Popham D.L., Weiss D.S., Ellermeier C.D. (2024). Identification of a family of peptidoglycan transpeptidases reveals that *Clostridioides difficile* requires noncanonical cross-links for viability. Proc. Natl. Acad. Sci. USA.

[17] Coullon H., Rifflet A., Wheeler R., Janoir C., Boneca I.G., Candela T. (2018). *N*-Deacetylases required for muramic-delta-lactam production are involved in *Clostridium difficile* sporulation, germination, and heat resistance. J. Biol. Chem..

[18] Kuru E., Hughes H.V., Brown P.J., Hall E., Tekkam S., Cava F., de Pedro M.A., Brun Y.V., VanNieuwenhze M.S. (2012). In Situ probing of newly synthesized peptidoglycan in live bacteria with fluorescent D-amino acids. Angew. Chem. Int. Ed. Engl..

[19] Kuru E., Radkov A., Meng X., Egan A., Alvarez L., Dowson A., Booher G., Breukink E., Roper D.I., Cava F. (2019). Mechanisms of Incorporation for D-Amino Acid Probes That Target Peptidoglycan Biosynthesis. ACS Chem. Biol..

[20] Toth M., Stewart N.K., Smith C., Vakulenko S.B. (2018). Intrinsic Class D beta-Lactamases of *Clostridium difficile*. mBio.

[21] Lam H., Oh D.C., Cava F., Takacs C.N., Clardy J., de Pedro M.A., Waldor M.K. (2009). D-amino acids govern stationary phase cell wall remodeling in bacteria. Science.

[22] Sutterlin L., Edoo Z., Hugonnet J.E., Mainardi J.L., Arthur M. (2018). Peptidoglycan Cross-Linking Activity of l,d-Transpeptidases from Clostridium difficile and Inactivation of These Enzymes by beta-Lactams. Antimicrob. Agents Chemother..

[23] Popham D.L., Bernhards C.B. (2015). Spore Peptidoglycan. Microbiol. Spectr..

[24] Fimlaid K.A., Bond J.P., Schutz K.C., Putnam E.E., Leung J.M., Lawley T.D., Shen A. (2013). Global analysis of the sporulation pathway of *Clostridium difficile*. PLoS Genet..

[25] Saujet L., Pereira F.C., Serrano M., Soutourina O., Monot M., Shelyakin P.V., Gelfand M.S., Dupuy B., Henriques A.O., Martin-Verstraete I. (2013). Genome-wide analysis of cell type-specific gene transcription during spore formation in *Clostridium difficile*. PLoS Genet..

[26] Soutourina O., Dubois T., Monot M., Shelyakin P.V., Saujet L., Boudry P., Gelfand M.S., Dupuy B., Martin-Verstraete I. (2020). Genome-Wide Transcription Start Site Mapping and Promoter Assignments to a Sigma Factor in the Human Enteropathogen *Clostridioides difficile*. Front. Microbiol..

[27] Sauvage E., Kerff F., Terrak M., Ayala J.A., Charlier P. (2008). The penicillin-binding proteins: structure and role in peptidoglycan biosynthesis. FEMS Microbiol. Rev..

[28] Triboulet S., Dubée V., Lecoq L., Bougault C., Mainardi J.L., Rice L.B., Ethève-Quelquejeu M., Gutmann L., Marie A., Dubost L. (2013). Kinetic features of L,D-transpeptidase inactivation critical for beta-lactam antibacterial activity. PLoS One.

[29] Mainardi J.L., Hugonnet J.E., Rusconi F., Fourgeaud M., Dubost L., Moumi A.N., Delfosse V., Mayer C., Gutmann L., Rice L.B., Arthur M. (2007). Unexpected inhibition of peptidoglycan LD-transpeptidase from *Enterococcus faecium* by the beta-lactam imipenem. J. Biol. Chem..

[30] Dubee V., Arthur M., Fief H., Triboulet S., Mainardi J.L., Gutmann L., Sollogoub M., Rice L.B., Etheve-Quelquejeu M., Hugonnet J.E. (2012). Kinetic analysis of *Enterococcus faecium* L,D-transpeptidase inactivation by carbapenems. Antimicrob. Agents Chemother..

[31] Sacco M.D., Wang S., Adapa S.R., Zhang X., Lewandowski E.M., Gongora M.V., Keramisanou D., Atlas Z.D., Townsend J.A., Gatdula J.R. (2022). A unique class of Zn(2+)-binding serine-based PBPs underlies cephalosporin resistance and sporogenesis in *Clostridioides difficile*. Nat. Commun..

[32] Dingle K.E., Freeman J., Didelot X., Quan T.P., Eyre D.W., Swann J., Spittal W.D., Clark E.V., Jolley K.A., Walker A.S. (2023). Penicillin Binding Protein Substitutions Cooccur with Fluoroquinolone Resistance in Epidemic Lineages of Multidrug-Resistant *Clostridioides difficile*. mBio.

[33] Alabdali Y.A.J., Oatley P., Kirk J.A., Fagan R.P. (2021). A cortex-specific penicillin-binding protein contributes to heat resistance in *Clostridioides difficile* spores. Anaerobe.

[34] Hussain H.A., Roberts A.P., Mullany P. (2005). Generation of an erythromycin-sensitive derivative of *Clostridium difficile* strain 630 (630Deltaerm) and demonstration that the conjugative transposon Tn916DeltaE enters the genome of this strain at multiple sites. J. Med. Microbiol..

[35] Peltier J., Hamiot A., Garneau J.R., Boudry P., Maikova A., Hajnsdorf E., Fortier L.C., Dupuy B., Soutourina O. (2020). Type I toxin-antitoxin systems contribute to the maintenance of mobile genetic elements in *Clostridioides difficile*. Commun. Biol..

[36] Galaxy Community (2022). The Galaxy platform for accessible, reproducible and collaborative biomedical analyses: 2022 update. Nucleic Acids Res..

[37] Rueden C.T., Schindelin J., Hiner M.C., DeZonia B.E., Walter A.E., Arena E.T., Eliceiri K.W. (2017). ImageJ2: ImageJ for the next generation of scientific image data. BMC Bioinf..

[38] Ducret A., Quardokus E.M., Brun Y.V. (2016). MicrobeJ, a tool for high throughput bacterial cell detection and quantitative analysis. Nat. Microbiol..

[39] Pino L.K., Searle B.C., Bollinger J.G., Nunn B., MacLean B., MacCoss M.J. (2020). The Skyline ecosystem: Informatics for quantitative mass spectrometry proteomics. Mass Spectrom. Rev..

[40] Bouillaut L., McBride S.M., Sorg J.A. (2011). Genetic manipulation of *Clostridium difficile*. Curr. Protoc. Microbiol..

[41] Permpoonpattana P., Tolls E.H., Nadem R., Tan S., Brisson A., Cutting S.M. (2011). Surface layers of *Clostridium difficile* endospores. J. Bacteriol..

[42] Glauner B., Höltje J.V., Schwarz U. (1988). The composition of the murein of *Escherichia coli*. J. Biol. Chem..

[43] Peltier J., Soutourina O. (2017). Identification of c-di-GMP-Responsive Riboswitches. Methods Mol. Biol..

[44] Criscuolo A., Brisse S. (2013). AlienTrimmer: a tool to quickly and accurately trim off multiple short contaminant sequences from high-throughput sequencing reads. Genomics.

[45] Langmead B., Salzberg S.L. (2012). Fast gapped-read alignment with Bowtie 2. Nat. Methods.

[46] Kanehisa M., Sato Y., Morishima K. (2016). BlastKOALA and GhostKOALA: KEGG Tools for Functional Characterization of Genome and Metagenome Sequences. J. Mol. Biol..

