# Supplementary material for: The l,d-transpeptidation pathway is inhibited by antibiotics of the β-lactam class in Clostridioides difficile
Source: iScience. 2025 Mar 16;28(4):112227. doi: 10.1016/j.isci.2025.112227 (PMC11986978; doi:10.1016/j.isci.2025.112227)
Supplement: Document S1. Figures S1–S7 and Tables S2–S5 [file mmc1.pdf]

iScience, Volume *28*

Supplemental information

The L,D-transpeptidation pathway is inhibited by antibiotics of the β-lactam class in *Clostridioides difficile*

Ana M. Oliveira Paiva, Pascal Courtin, Glenn Charpentier, Imane Oueled-Chama, Olga Soutourina, Marie-Pierre Chapot-Chartier, and Johann Peltier

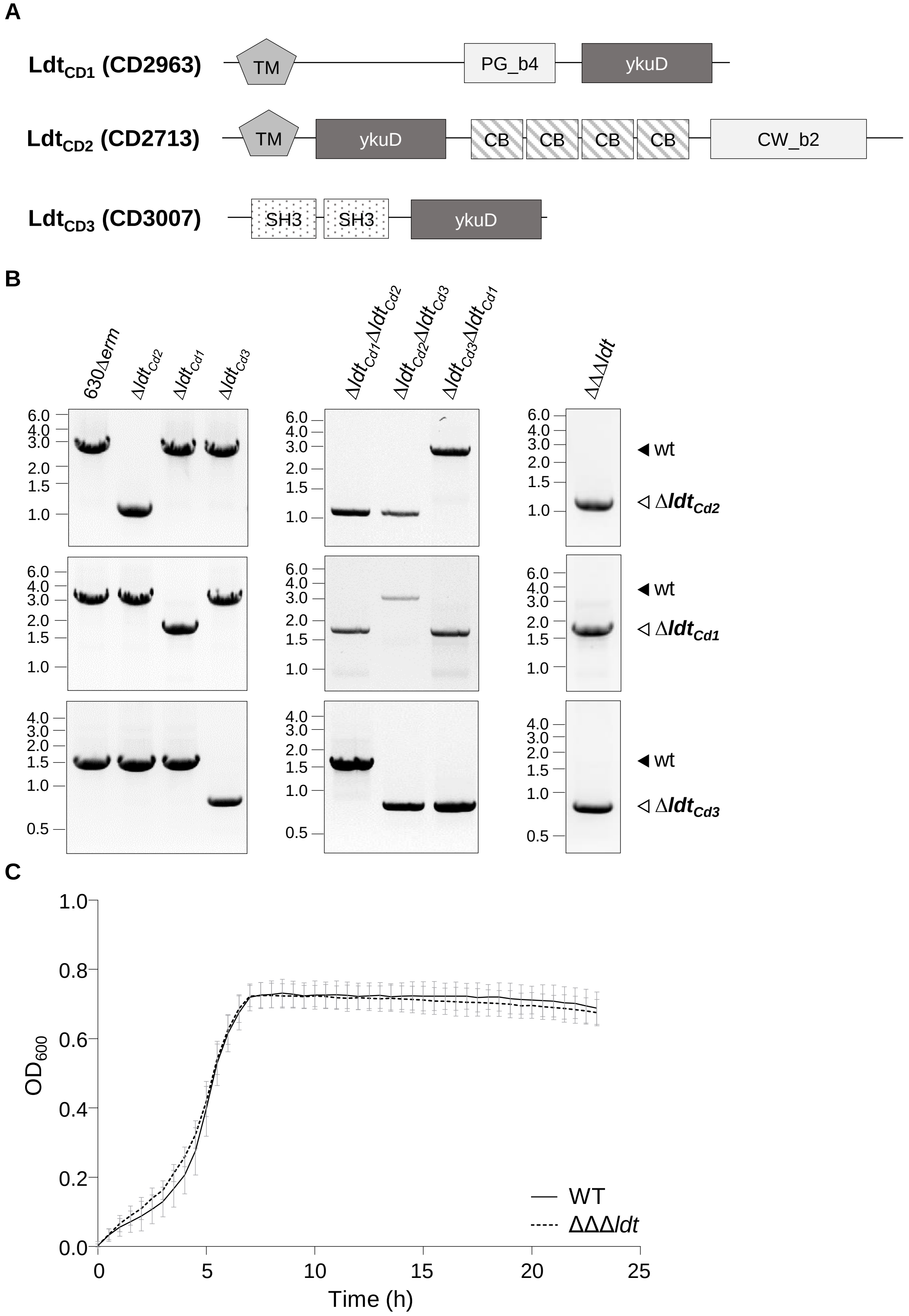

Figure S1 - Impact of a triple mutant of the LDT-encoding genes on *C. difficile* growth.

(A) Domain composition of the L.D-transpeptidases Ldt$_{Cd1}$ (CD2963), Ldt$_{Cd2}$ (CD2713) and Ldt$_{Cd3}$ (CD3007) from *C. difficile*. The different conserved domains are represented: transmembrane region (TM in grey), peptidoglycan and cell-wall binding domain (PG_b4 and CW_b2. in light grey), LDT catalytic domain (YkuD in dark grey), cholin-binding domain (CB in stripes) and the SH3 domain associated with a variety of intracellular or membrane-associated proteins (dots).

(B) PCR amplification from *C. difficile* 630Δ*erm* wild-type (WT), ΔΔΔ*ldt*, Δ*ldt$_{Cd1}$*, Δ*ldt$_{Cd2}$*, Δ*ldt$_{Cd3}$*, Δ*ldt$_{Cd1}$*Δ*ldt$_{Cd2}$*, Δ*ldt$_{Cd1}$*Δ*ldt$_{Cd3}$* and Δ*ldt$_{Cd2}$*Δ*ldt$_{Cd3}$*, with specific primers to the locus region of *ldt$_{Cd1}$*, *ldt$_{Cd2}$* and *ldt$_{Cd3}$* to verify the different alleles.

(C) Growth curves of *C. difficile* WT and ΔΔΔ*ldt* strains in BHI medium. Means and SD are shown; *n* = 3 independent experiments.

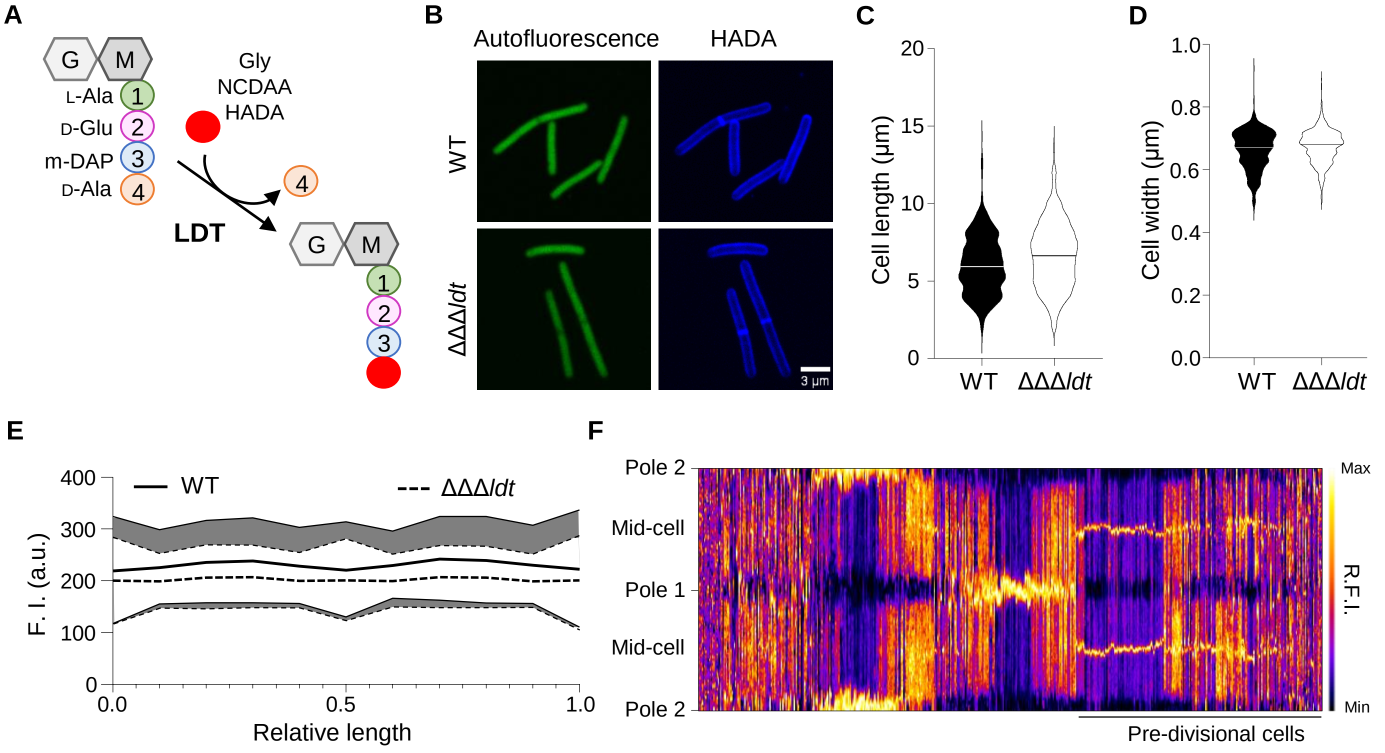

Fig. S2. Analysis of cell morphology and HADA incorporation in *C. difficile* 630*Δerm* and ΔΔΔ*ldt*.
(A) Schematic representation of the amino-acid exchange reaction catalyzed by LDTs. LDTs can catalyze the exchange of the terminal D-Ala of a PG tetrapeptide stem by a glycine, a non-canonical aminoacid (NCDAA) or the HADA analogue. G: N-acetylglucosamine, M: N-acetylmuramic acid.
(B) Cells were collected at an OD_{600nm} of 0.6 and stained with the fluorescent D-amino-acid HADA for 10 min. Cells were imaged on the green channel for the autofluorescence (excitation, 488nm) and on the blue channel for HADA (excitation, 405nm). Scalebar=3 µm. Pictures were treated equally and are representative of 3 independent experiments.
(C) Scatter plot showing cell length of 630*Δerm* and ΔΔΔ*ldt* strains with the median of each distribution indicated by a black line.
(D) Scatter plot showing cell width of 630*Δerm* and ΔΔΔ*ldt* strains with the median of each distribution indicated by a black line.
(E) Average contour fluorescence intensity (HADA distribution). Standard deviation is represented by dashed lines.
(F) Analysis of the contour relative fluorescence intensity (HADA distribution) in *C. difficile* 630*Δerm* cells. Cells were grouped by fluorescent distribution and pre-divisional cells were identified. Pole 1 and 2, as well as mid-cell are shown. Scale of fluorescent intensity is depicted.
For panels (C) to (F), 2 independent experiments were analysed for *C. difficile* 630*Δerm* (grey, *n*=544) and ΔΔΔ*ldt* (orange, *n*=878).

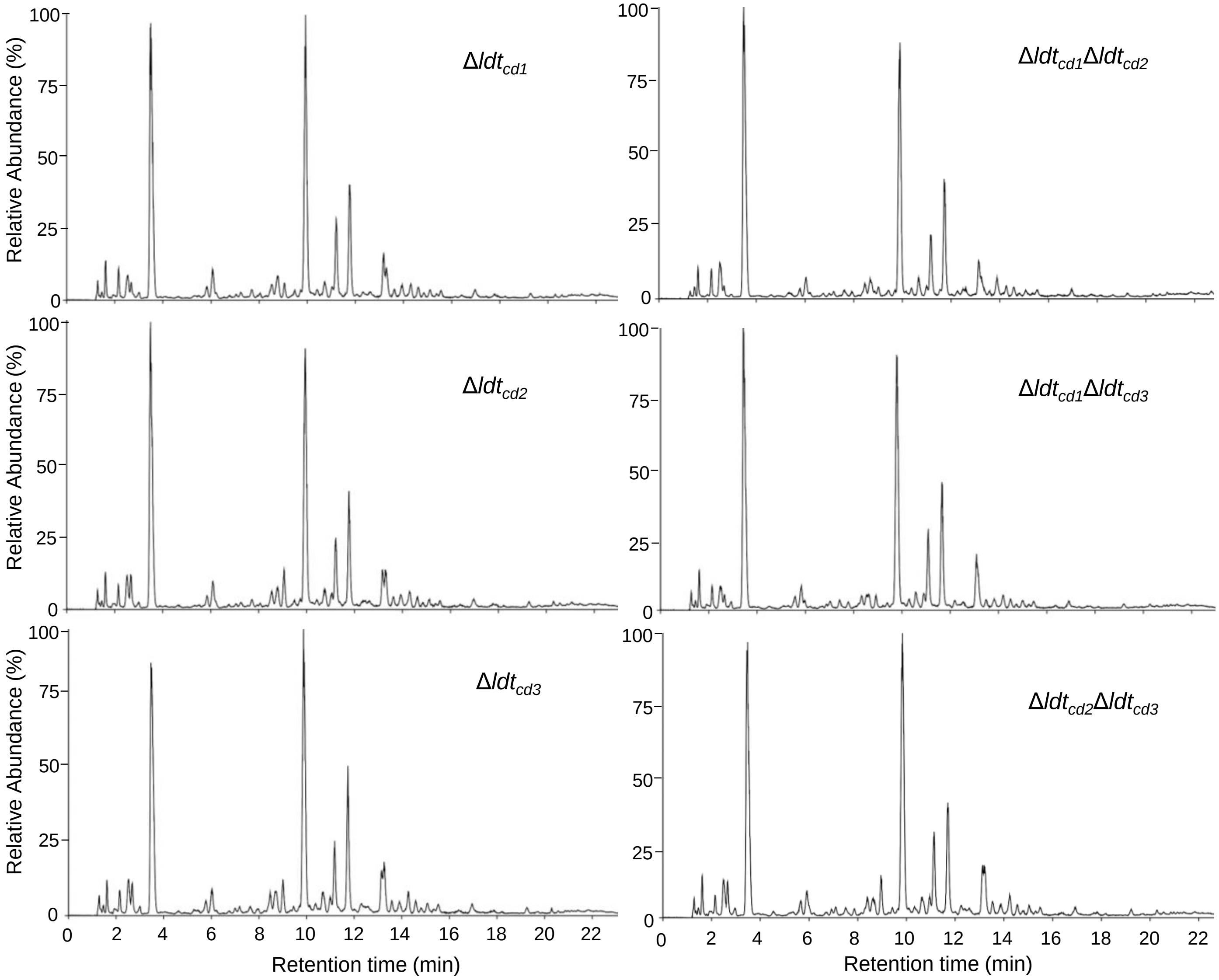

Figure S3 - Impact of *ldt* single and double mutants on the PG structure of *C. difficile* vegetative cells. LC-MS analysis (TIC chromatogram) of muropeptides from vegetative cells of *C. difficile* Δ*ldt*$_{cd1}$, Δ*ldt*$_{cd2}$, Δ*ldt*$_{cd3}$, Δ*ldt*$_{cd1}$Δ*ldt*$_{cd2}$, Δ*ldt*$_{cd1}$Δ*ldt*$_{cd3}$ and Δ*ldt*$_{cd2}$Δ*ldt*$_{cd3}$ strains. See also Table S1 for the structure of all identified muropeptides. Data are representative of two independent experiments.

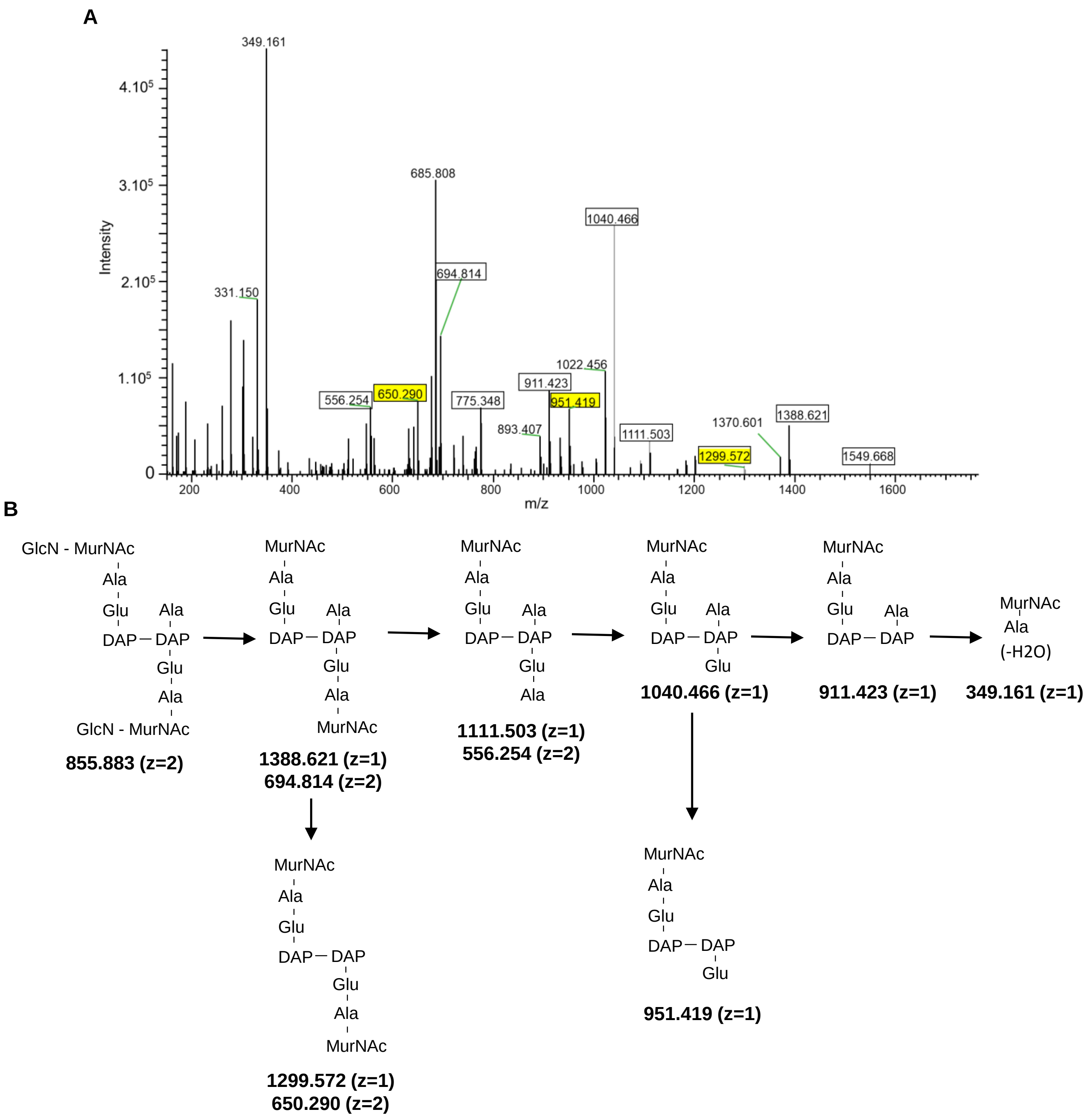

Figure S4: MS/MS analysis of the second muropeptide dimer from the spore cortex of *C. difficile* wild type.

(A) MS/MS analysis of the ion (m/z = 855.883 with z=2) corresponding to the peak with a retention time of 8.75 min in Fig. 4A and Table S2. Fragments (squared) were detected as $[M+H]^+$ (z=1) or $[M+2H]^{2+}$ (z=2) adducts. Fragments resulting from loss of a water molecule are not squared. In yellow, the fragments allowing the assignment of the 3-3 cross-link.

(B) Structures inferred from the MS/MS analysis are presented. The loss of one alanine from the C-terminal end of different ions (mass loss of 89.048) establishes the presence of a 3→3 crosslink.

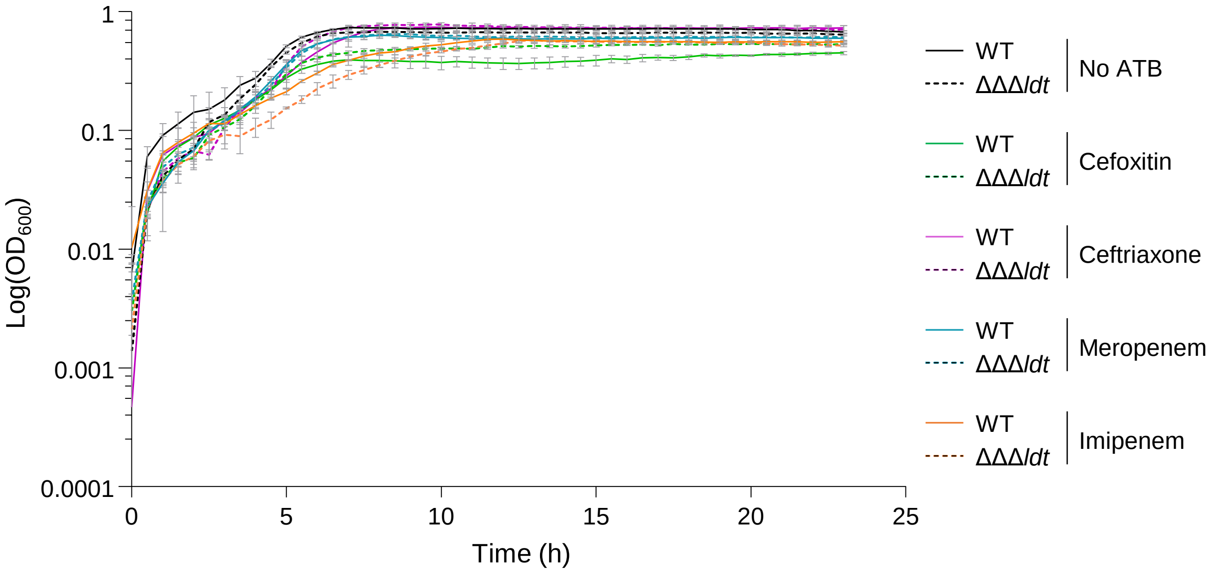

Figure S5 - Growth curve of of *C. difficile* 630Δ*erm* wild-type (WT) and ΔΔΔ*ldt* strains in the presence of sub-MIC of β-lactam antibiotics in BHI medium. Means and SD are shown; *n* = 3 independent experiments.

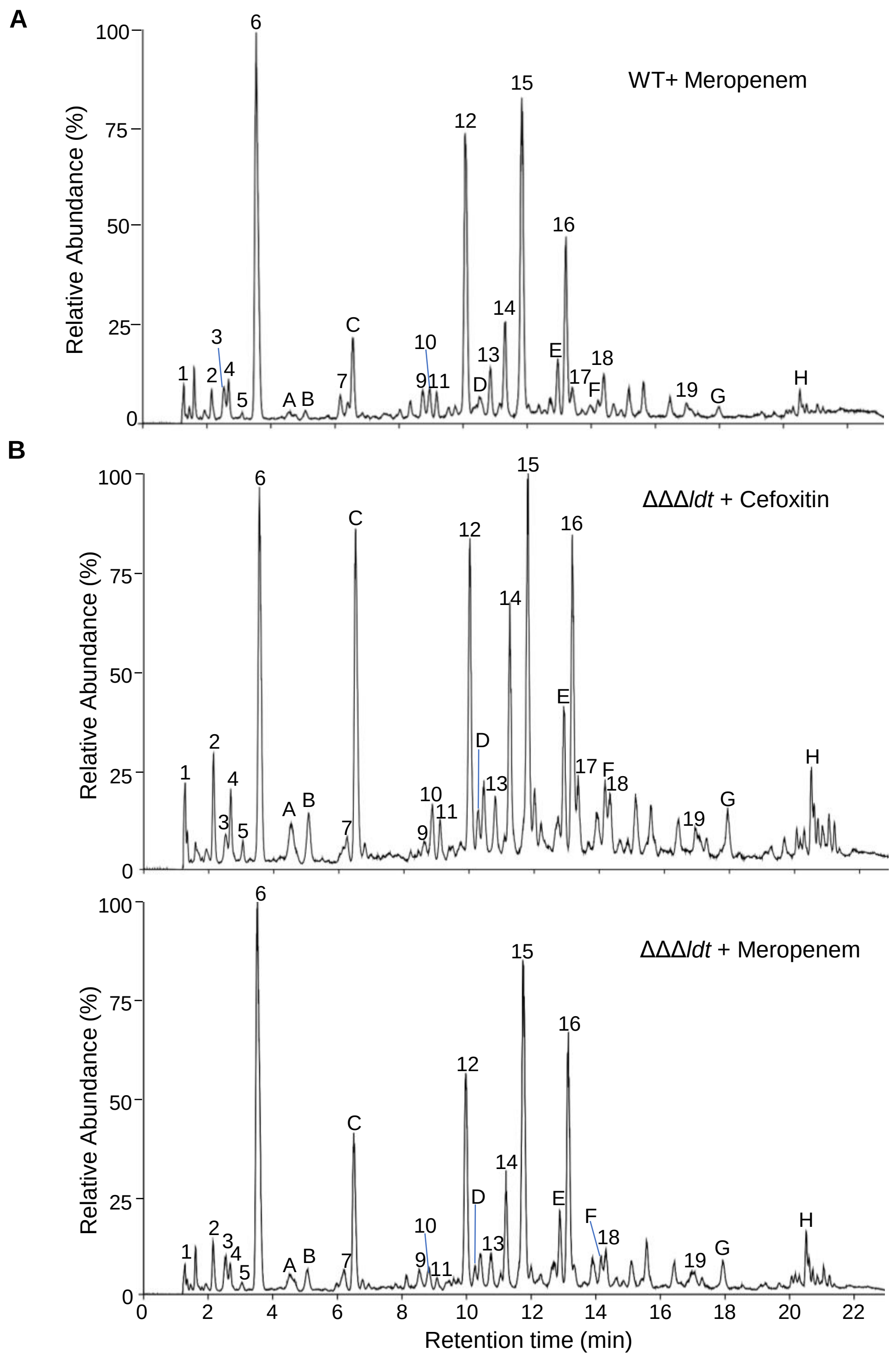

Figure S6 - LC-MS analysis (TIC chromatogram) of muropeptides of *C. difficile WT* (A) *and* ΔΔΔ*ldt* (B) strain grown in the presence of subinhibitory concentrations of cefoxitin or meropenem. Peak labels refer to Table S1. New major peaks observed in the presence of the antibiotics are labelled with letters. See also Table S1 for the structure of all identified muropeptides. Data are representative of three independent experiments.

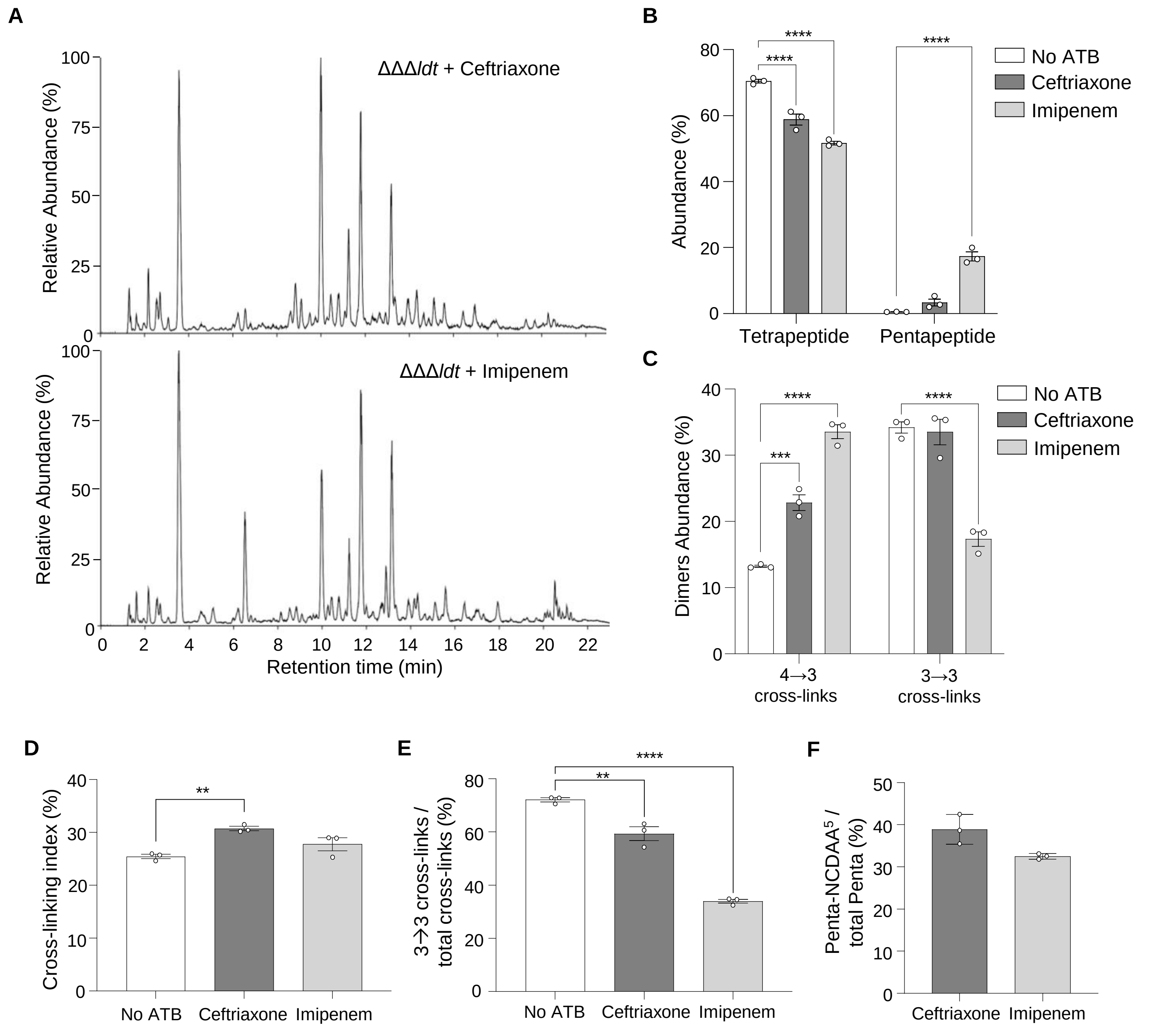

Figure S7 - Impact of the cephalosporin ceftriaxone and the carbapenem imipenem on the PG structure of vegetative cells of *C. difficile* ΔΔΔ*ldt*.

(A) LC-MS analysis (TIC chromatogram) of muropeptides from vegetative cells of *C. difficile* ΔΔΔ*ldt* strain grown in the presence of subinhibitory concentrations of ceftriaxone or imipenem. See also Table S2 for the structure of all identified muropeptides. Data are representative of three independent experiments.

(B) Abundance of muropeptides with a tetrapeptide (Tetrapep.) or a pentapeptide (Pentapep.) stem in the PG of *C. difficile* ΔΔΔ*ldt* strain grown in absence of antibiotics (no ATB) or in the presence of ceftriaxone or imipenem.

(C) Abundance of muropeptide dimers with a 4→3 or a 3→3 crosslink relative to the total muropeptides in the PG of *C. difficile* ΔΔΔ*ldt* strain grown in the absence of antibiotics (no ATB) or in the presence of ceftriaxone or imipenem.

(D) Cross-linking index of the PG of *C. difficile* ΔΔΔ*ldt* strain grown in absence of antibiotics (no ATB) or in the presence of ceftriaxone or imipenem.

(E) Abundance of muropeptide dimers with a 3→3 crosslink relative to the total crosslinks in dimers in the PG of *C. difficile* ΔΔΔ*ldt* strain grown in the absence of antibiotics (no ATB) or in the presence of ceftriaxone or imipenem.

(F) Abundance of muropeptides with a pentapeptide stem ending with a non-canonical D-amino-acid (NCDAA: Gly, Phe, Leu or Val) relative to the total pentapeptide muropeptides in the ΔΔΔ*ldt* strain grown in the presence of ceftriaxone (ceftri.) or imipenem (imip.).

All graphs represent mean ± SD and include individual data points; $n = 3$ independent experiments. **$P \leq 0.01$, ***$P \leq 0.001$ and ****$P \leq 0.0001$ by a two-way ANOVA (B and C) or a one-way ANOVA (D and E), followed by a Dunnett's multiple comparisons test.

Table S2 - Structures, molecular masses and proportions of muropeptides from spores of *C. difficile* 630Δ*erm* and ΔΔΔl*dt* strains.

RT[a] (min)	Muropeptide	Peak #[b]	m/z [M+H+]		Abundance ± SD (%)[d]	
			Observed[c]	Calculated	630Δ*erm*	*ΔΔΔldt*
**No cross-link**					**96.989 ± 0.627**	**95.633 ± 0.215**
1.24	GM	1	499.2136	499.2139	10.125 ± 6.027	9.232 ± 6.29
1.36	GM (nr)	2	497.1975	497.1983	5.473 ± 2.831	5.402 ± 2.119
1.67	GM (nr)	3	497.1970	497.1983	0.974 ± 0.419	0.944 ± 0.275
1.87	GM (nr)	4a	497.1980	497.1983	1.335 ± 0.67	1.189 ± 0.381
1.87	GM-GM	4b	977.4001	977.3938	0.505 ± 0.136	0.512 ± 0.266
3.55	GM-Tetra	5	942.4172	942.4155	8.098 ± 1.037	7.539 ± 0.785
4.2	GM-Tetra (deAc)	6	900.4064	900.4049	7.644 ± 1.649	8.38 ± 0.664
5.04	GMLred-GM (deAc)	7	861.3795	861.3828	3.425 ± 3.498	2.772 ± 2.745
5.39	GMLred-GM	8	903.3884	903.3934	2.172 ± 0.606	3.14 ± 2.249
6.09	GMLred-GM (deAc) (nr)	9	859.3647	859.3671	1.232 ± 0.074	1.25 ± 0.186
6.78	GM-GMLred-GM (deAc) (nr)	10	1337.5505	1337.5470	0.864 ± 0.01	0.84 ± 0.217
7.13	GM-GMLred-GM (deAc) (nr)		1337.5491	1337.5470	0.249 ± 0.026	0.237 ± 0.111
7.23	GMLred-GM-Tetra (deAc)	11a	1304.5859	1304.5844	2.29 ± 1.743	1.993 ± 1.059
7.28	GMLred-GM-Tetra (deAc x2)	11b	1262.5721	1262.5738	1.482 ± 1.155	1.338 ± 0.814
7.42	GMLred-GM-Tetra	12	1346.5947	1346.5950	2.502 ± 1.442	2.144 ± 1.077
7.85	GML-GM (deAc) (nr)	13	873.3471	873.3464	2.857 ± 1.837	3.301 ± 1.173
8.08	GML-GM (deAc) (nr)		873.3405	873.3464	0.67 ± 0.343	0.61 ± 0.437
8.7	GML-GM-Tetra (deAc)	15a	1318.5667	1318.5636	3.388 ± 0.296	3.481 ± 0.709
8.78	GM-GML-GM (deAc) (nr)	15b	1351.5245	1351.5263	2.156 ± 1.308	2.143 ± 0.966
9.07	GM-GML-GM (deAc) (nr)		1351.5271	1351.5263	0.633 ± 0.435	0.551 ± 0.219
9.48	GML-GM-Tetra (deAc)	16	1318.5731	1318.5636	11.53 ± 4.235	12.918 ± 5.576
9.82	GML-GM (nr)	17	915.3595	915.3570	1.653 ± 1.303	1.692 ± 0.983
9.99	GML-GM-Tetra	18	1360.5767	1360.5743	3.955 ± 0.94	3.796 ± 0.864
10.15	GM-GML-GM-Tetra (deAc x2)		1754.7395	1754.7330	0.435 ± 0.064	0.469 ± 0.068
10.54	GML-GM-Di (deAc)	19	1075.4482	1075.4418	2.416 ± 0.463	2.314 ± 0.317
10.63	GM-GML-GM-Tetra (deAc x2)		1754.7395	1754.7330	0.237 ± 0.01	0.313 ± 0.055
10.8	GML-GM-GMLred-GM (deAc x2)		1715.7189	1715.7108	0.545 ± 0.37	0.537 ± 0.241
10.94	GM-Tri		871.3845	871.3784	0.128 ± 0.068	0.134 ± 0.062
11.17	GML-GM-GMLred-GM (deAc x2)		1715.7189	1715.7108	0.943 ± 0.688	0.797 ± 0.452
11.39	GML-GM-Di		1117.4617	1117.4524	0.708 ± 0.112	0.655 ± 0.042
11.69	GML-GM-GMLred-GM (deAc)		1757.7319	1757.7138	0.887 ± 0.309	0.741 ± 0.405
11.83	GML-GM-GMLred-GM (deAc x2) (nr)		1713.6999	1713.6952	0.397 ± 0.047	0.46 ± 0.093
11.87	GML-GM-GMLred-GM (deAc)		1757.7319	1757.7214	0.892 ± 0.396	0.627 ± 0.366
11.97	GML-GM-GMLred-GM (deAc x2) (nr)		1713.6999	1713.6952	0.567 ± 0.139	0.587 ± 0.198
12.17	GML-GM-GMLred-GM (deAc x2) (nr)		1713.6999	1713.6952	0.399 ± 0.27	0.221 ± 0.045
12.21	GML-GM-GML-GM (deAc x2)	20	1729.6923	1729.6901	1.217 ± 0.302	1.226 ± 0.428
12.42	GML-GMLred-GM (deAC) (nr)		1277.5277	1277.5259	0.405 ± 0.239	0.403 ± 0.096
12.49	GML-GM-GMLred-GM-Tetra(-deAc x2)	21	2158.9253	2158.9124	1.091 ± 0.191	1.123 ± 0.261
12.55	GML-GM-GMLred-GM (deAc) (nr)		1755.7135	1755.7058	0.307 ± 0.122	0.316 ± 0.16
12.97	GML-GML-GM (deAC)		1293.5210	1293.5208	0.776 ± 0.429	0.66 ± 0.188
13.01	GML-GMLred-GM (deAC) (nr)		1277.5357	1277.5259	0.592 ± 0.169	0.552 ± 0.119
13.3	GML-GM-GML-GM (deAc x2) (nr)		1727.6805	1727.6744	1.451 ± 1.107	1.533 ± 0.897
13.3	GML-GM-GML-GM (deAc x2)		1729.6923	1729.6901	0.591 ± 0.426	0.546 ± 0.3
13.38	GML-GM-GML-GM (deAc)	22a	1771.7025	1771.7007	1.519 ± 0.696	1.319 ± 0.274
13.4	GML-GM-GMLred-GM (deAc) (nr)	22b	1755.7125	1755.7058	0.427 ± 0.279	0.373 ± 0.175
13.51	GML-GM-GML-GM (deAc x2) (nr)		1727.6859	1727.6744	0.69 ± 0.434	0.723 ± 0.382
13.73	GML-GM-GML-GM-Tetra(-deAc x2)	23	2172.9071	2172.8917	0.988 ± 0.632	0.822 ± 0.345
14.55	GML-GML-GM (deAc) (nr)	24	1291.5117	1291.5005	1.624 ± 1.165	1.508 ± 0.769
14.89	GML-GM-GML-GM (deAc) (nr)	25	1769.6971	1769.6850	1.344 ± 1.086	1.032 ± 0.68
15.2	GML-GM-GML-GM-GMLred-GM (deAc x2)		2612.0534	2612.0495	0.145 ± 0.1	0.148 ± 0.022
15.53	GML-GM-GML-GM-GML-GM (deAc x2)		2626.0343	2626.0287	0.001 ± 0	0.001 ± 0
15.83	GML-GM-GML-GM-GML-GM-Tetra (deAc x3)		3027.2465	3027.2197	0.053 ± 0.035	0.09 ± 0.001
**3→3 cross-link**					**3,011 ± 0,627**	**4,367 ± 0,215**
7.99	GM-Tri →GM-Tetra (deAc x2)	14	1710.7663	1710.7544	1.668 ± 0.405	2.391 ± 0.272
8.75	GM-Tri →GM-Tetra (deAc x2)	15c	1710.7611	1710.7544	1.343 ± 0.224	1.976 ± 0.07

Abbreviations: RT: retention time; SD: standard deviation; G: *N*-acetylglucosamine; M: *N*-acetylmuramic acid; ML: muramic-δ-lactam; MLred: reduced muramic-δ-lactam; Di: dipeptide (L-Ala-D-Glu); Tri: tripeptide (L-Ala-D-Glu-A2pm); Tetra: tetrapeptide (L-Ala-D-Glu-A2pm-D-Ala) ; deAc: deacetylation of *N*-acetylglucosamine. MurNAc residues at the reducing end of muropeptides were reduced by $NaBH_4$ treatment, unless indicated (nr, non reduced).

[a] Retention times correspond to the LC-MS/MS separation of muropeptides from *C. difficile* 630Δ*erm* shown in Fig. 4A.

[b] Peak numbers refer to Fig. 4A. A few peaks contain two or three muropeptides named a and b or a, b and c, respectively. Only major peaks are numbered.

[c] Observed masses correspond to the LC-MS/MS separation of muropeptides from *C. difficile* 630Δ*erm* shown in Fig. 4A.

[d] The average abundances and the standard deviations from three biological replicates are shown.

Table S3 – Minimum inhibitory concentration (MIC) of β-lactam antibiotics against *C. difficile* 630Δ*erm* and triple mutant *ΔΔΔldt*.

**MIC (µg/mL)**	**630Δ*erm***	***ΔΔΔldt***
Amoxicillin	8	8
Oxacillin	128	128
Ceftriaxone	64	64
Cefoxitine	128	128
Imipenem	4	4
Meropenem	2	2

Determination was performed as described in material and methods in three independent assays.

Table S4 – Genes upregulated in ΔΔΔldt grown in presence of 32 µg/mL of ceftriaxone.

Gene	$\log_2$FoldChange[a]	Classification	*q* value[b]
CD630_SQ808	5.631	Antitoxin from type I toxin/antitoxin system	4.21E-04
CD630_Cdi1_5	5.448	c-di-GMP-I riboswitches	4.77E-04
CD630_28890	5.218	Toxin from type I toxin/antitoxin system	4.77E-04
CD630_09771	5.212	Toxin from type I toxin/antitoxin system	4.77E-04
CD630_Cdi1_4	5.173	c-di-GMP-I riboswitches	4.77E-04
CD630_12331	5.155	Toxin from type I toxin/antitoxin system	4.77E-04
CD630_n00150	5.112	Antitoxin from type I toxin/antitoxin system	4.77E-04
CD630_n00370	5.103	Antitoxin from type I toxin/antitoxin system	4.77E-04
CD630_25171	5.069	Toxin from type I toxin/antitoxin system	4.77E-04
CD630_04401	5.045	Toxin from type I toxin/antitoxin system	4.77E-04
CD630_29071	4.999	Toxin from type I toxin/antitoxin system	4.77E-04
CD630_n01000	4.99	Antitoxin from type I toxin/antitoxin system	4.77E-04
CD630_09562	4.977	Toxin from type I toxin/antitoxin system	4.77E-04
CD630_SQ1781	4.92	Antitoxin from type I toxin/antitoxin system	4.77E-04
CD630_n00830	4.904	ncRNA	4.77E-04
CD630_n00500	4.839	Antitoxin from type I toxin/antitoxin system	6.17E-04
CD630_22991	4.83	Toxin from type I toxin/antitoxin system	6.17E-04
CD630_SQ22	4.815	ncRNA	6.17E-04
CD630_n00400	4.791	CRISP	6.26E-04
CD630_15951	4.781	Unclassified: metabolism	6.26E-04
CD630_SQ1076	4.78	ncRNA	6.26E-04
CD630_s0020	4.709	ncRNA	6.62E-04
CD630_n00350	4.489	Antitoxin from type I toxin/antitoxin system	8.80E-04
CD630_12800	4.422	Metabolism	1.16E-03
CD630_33300	4.271	Mobile element	2.09E-03
CD630_10040 (fumB)	4.087	Metabolism	3.74E-03
CD630_s0570	4.016	ncRNA	3.74E-03
CD630_n00410	4.014	ncRNA	4.02E-03
CD630_23510 (grdB)	4.011	Unclassified: metabolism	4.40E-03
CD630_10910 (int1)	4.01	Mobile element	4.53E-03
CD630_n00430	3.949	ncRNA	4.66E-03
CD630_s0590	3.904	ncRNA	5.94E-03
CD630_n00420	3.903	ncRNA	6.60E-03
CD630_n00040	3.876	ncRNA	6.60E-03
CD630_05090	3.791	Mobile element	7.16E-03
CD630_01721 (fdx*A*)	3.73	Metal metabolism	7.18E-03
CD630_s0600	3.711	ncRNA	7.18E-03
CD630_04532	3.709	Mobile element	7.18E-03
CD630_33760	3.682	Mobile element	7.58E-03
CD630_33770 (mgtA1)	3.666	Unclassified: metabolism	7.58E-03
CD630_s0300	3.62	ncRNA	7.58E-03
CD630_SQ2397	3.595	ncRNA	7.59E-03

CD630_s0340	3.579	ncRNA	7.75E-03
CD630_Cdi1_11	3.564	c-di-GMP-I riboswitches	8.45E-03
CD630_s0310	3.558	ncRNA	8.45E-03
CD630_s0480	3.529	ncRNA	8.79E-03
CD630_33682	3.518	Unclassified	8.81E-03
CD630_n00600	3.492	CRISP	8.86E-03
CD630_Cdi1_9	3.487	c-di-GMP-I riboswitches	9.38E-03
CD630_23090	3.474	Unclassified	1.00E-02
CD630_27991	3.457	Unclassified	1.13E-02
CD630_36490 (rlmH)	3.429	Translation	1.17E-02
CD630_25621 (rpmB)	3.419	Translation	1.17E-02
CD630_33420	3.412	Mobile element	1.17E-02
CD630_s0450	3.387	ncRNA	1.23E-02
CD630_n00860	3.357	CRISP	1.24E-02
CD630_n00640	3.34	ncRNA	1.25E-02
CD630_s0010	3.312	ncRNA	1.25E-02
CD630_Cdi1_10	3.297	c-di-GMP-I riboswitches	1.28E-02
CD630_Cdi1_8	3.285	c-di-GMP-I riboswitches	1.32E-02
CD630_27280	3.275	Unclassified	1.34E-02
CD630_19903	3.267	Unclassified	1.34E-02
CD630_32520	3.264	Unclassified	1.43E-02
CD630_s0580	3.218	ncRNA	1.51E-02
CD630_11031	3.212	Mobile element	1.51E-02
CD630_15950 (cysE)	3.203	Biofilm formation	1.57E-02
CD630_14240	3.181	Unclassified	1.57E-02
CD630_n00990	3.163	CRISP	1.58E-02
CD630_11030	3.161	Mobile element	1.59E-02
**Gene**	**log$_2$FoldChange[a]**	**Classification**	***q* value[b]**
CD630_s0660	3.136	ncRNA	1.64E-02
CD630_s0170	3.134	ncRNA	1.72E-02
CD630_19430	3.111	Unclassified	1.79E-02
CD630_03110 (gerG)	3.105	Sporulation	1.85E-02
CD630_12570 (rplS)	3.072	Translation	1.85E-02
CD630_10980	3.022	Unclassified	2.08E-02
CD630_16930	3.019	Unclassified	2.08E-02
CD630_s0642	2.985	ncRNA	2.09E-02
CD630_s0200	2.976	ncRNA	2.09E-02
CD630_n00380	2.972	ncRNA	2.09E-02
CD630_12780 (iscR)	2.938	Metabolism	2.24E-02
CD630_19970	2.919	Unclassified	2.45E-02
CD630_11040	2.916	Mobile element	2.45E-02
CD630_s0260	2.91	ncRNA	2.51E-02
CD630_27270	2.879	Unclassified	2.52E-02
CD630_33750 (mgtC)	2.86	Protein families: signaling and cellular processes	2.52E-02
CD630_36051	2.854	Metal metabolism	2.52E-02
CD630_s0380	2.85	ncRNA	2.63E-02
CD630_03590	2.847	Mobile element	2.84E-02
CD630_s0410	2.844	ncRNA	2.88E-02
CD630_14250	2.843	Protein families: signaling and cellular processes	2.88E-02
CD630_08240	2.842	ABC transporters	3.07E-02
CD630_s0560	2.821	ncRNA	3.07E-02
CD630_s0440	2.818	ncRNA	3.07E-02
CD630_13160 (rpsO)	2.79	Translation	3.12E-02
CD630_s0400	2.779	ncRNA	3.16E-02
CD630_s0390	2.778	ncRNA	3.24E-02
CD630_s0530	2.755	ncRNA	3.27E-02
CD630_21950 (ftnA)	2.739	Unclassified: metabolism	3.37E-02
CD630_13920	2.736	Unclassified	3.40E-02
CD630_s0230	2.716	ncRNA	3.40E-02
CD630_33310	2.712	Mobile element	3.42E-02
CD630_30730	2.7	Protein families: signaling and cellular processes	3.42E-02
CD630_00180 (recR)	2.666	Replication and repair	3.48E-02
CD630_03580	2.654	Mobile element	3.51E-02
CD630_13000 (pepA)	2.647	Aminoacid metabolism	3.51E-02
CD630_13550 (cspB)	2.631	Unclassified: genetic information processing	3.53E-02
CD630_21680 (hcp)	2.631	Energy metabolism	3.53E-02
CD630_00150 (*tadA*)	2.619	Translation	3.56E-02
CD630_s0350	2.616	ncRNA	3.68E-02
CD630_08210	2.609	Signal transduction	3.72E-02
CD630_11761 (rpmF)	2.588	Translation	3.80E-02
CD630_23100 (cspD)	2.579	Unclassified: genetic information processing	3.83E-02
CD630_s0240	2.535	ncRNA	3.96E-02

CD630_19401	2.522	Unclassified	3.96E-02
CD630_04980	2.504	Mobile element	3.99E-02
CD630_04530	2.502	Metal metabolism	4.21E-02
CD630_08200	2.502	Signal transduction	4.33E-02
CD630_26160	2.49	Unclassified	4.33E-02
CD630_11320 (copZ)	2.478	Metal metabolism	4.45E-02
CD630_14130	2.465	Unclassified	4.45E-02
CD630_02510 (fliI)	2.459	Cell motility	4.56E-02
CD630_34861 (rpmE)	2.455	Translation	4.58E-02
CD630_n00620	2.438	ncRNA	4.64E-02
CD630_05000	2.422	Mobile element	4.66E-02
CD630_06070	2.397	Mobile element	4.66E-02
CD630_10620 (acpP)	2.385	Metabolism	4.75E-02
CD630_11241	2.382	Unclassified	4.84E-02
CD630_n00440	2.378	ncRNA	4.84E-02
CD630_25060	2.375	Unclassified	4.90E-02
CD630_n00900	2.331	ncRNA	4.90E-02
CD630_10590 (thlA1)	2.328	Metabolism	4.90E-02
CD630_08920 (cspA)	2.323	Germination	4.90E-02
CD630_17451 (feoA3)	2.23	Metal metabolism	4.92E-02

[a]The fold changes listed are averages from two biological replicates. Fold changes signify the differential expression in *C. difficile* ΔΔΔ*ldt* compared with the 630Δ*erm* wild-type strain.
[b]The *q* value is an adjusted *P* value, taking into account the false discovery rate.

Table S5 – Oligonucleotides used in this study.

**Primer**	**Sequence (5’ to 3’)**	**Use**
oAP7	ttttttgttaccctaagtttccaagtagatatagaacatcttc	5’ left arm for *CD2963* deletion
oAP8	tacctggttcctcttttccatcaatcataattaaatc	3’ left arm for *CD2963* deletion
oAP9	tggaaaagaggaaccaggtacaccaattattttatac	5’ right arm for *CD2963* deletion
oAP10	agattatcaaaaaggagtttggtttttaaataccattattttcaataattataatttaaag	3’ right arm for *CD2963* deletion
oAP11	ggggtgatttataatagtcattttgtc	5’ *CD2963* deletion screening
oAP12	ctacctcattgttaaagtataaacagc	3’ *CD2963* deletion screening
oAP3	ttttttgttaccctaagtttgctatttctaaaccttctctatag	5’ left arm for *CD2713* deletion
oAP4	gtctaaaaaaggataaattaatggatattcttgcaaaatag	3’ left arm for *CD2713* deletion
oAP5	ttaatttatcctttttagactttttttcactataac	5’ right arm for *CD2713* deletion
oAP6	agattatcaaaaaggagtttgaattaatgagagaatatggtg	3’ right arm for *CD2713* deletion
oAP1	cactttgcagctttaaaagc	5’ *CD2713* deletion screening
oAP2	cttactcaccctcttaacatatgatg	3’ *CD2713* deletion screening
oAP13	tttttgttaccctaagtttggttttcaagtaaagaattaacaag	5’ left arm for *CD3007* deletion
oAP14	ctgttgttgtatctctatatttttaatatttagtttagttaatttcac	3’ left arm for *CD3007* deletion
oAP15	aatatagagatacaacaacagttataattcattaag	5’ right arm for *CD3007* deletion
oAP53	gtttttgttaccctaagtttctgatgaagacagacatgtaaag	3’ right arm for *CD3007* deletion
oAP17	gtggctagaggtataattgaagag	5’ *CD3007* deletion screening
oAP18	cgtagctataactactactgctg	3’ *CD3007* deletion screening
